# Heparan Sulfated Glypican-4 Is Released from Astrocytes by Proteolytic Shedding and GPI-Anchor Cleavage Mechanisms

**DOI:** 10.1523/ENEURO.0069-21.2021

**Published:** 2021-08-06

**Authors:** Kevin Huang, Sungjin Park

**Affiliations:** 1 Department of Neurobiology, University of Utah School of Medicine, Salt Lake City, UT 84112; 2 Interdepartmental Program in Neuroscience, University of Utah, Salt Lake City, UT 84112

**Keywords:** ADAM9, astrocyte, glypican-4, GPI anchor, heparan sulfate, synaptogenesis

## Abstract

Astrocytes provide neurons with diffusible factors that promote synapse formation and maturation. In particular, glypican-4/GPC4 released from astrocytes promotes the maturation of excitatory synapses. Unlike other secreted factors, GPC4 contains the C-terminal GPI-anchorage signal. However, the mechanism by which membrane-tethered GPC4 is released from astrocytes is unknown. Using mouse primary astrocyte cultures and a quantitative luciferase-based release assay, we show that GPC4 is expressed on the astrocyte surface via a GPI-anchorage. Soluble GPC4 is robustly released from the astrocytes largely by proteolytic shedding and, to a lesser extent, by GPI-anchor cleavage, but not by vesicular release. Pharmacological, overexpression, and loss of function screens showed that ADAM9 in part mediates the release of GPC4 from astrocytes. The released GPC4 contains the heparan sulfate side chain, suggesting that these release mechanisms provide the active form that promotes synapse maturation and function. Overall, our studies identified the release mechanisms and the major releasing enzyme of GPC4 in astrocytes and will provide insights into understanding how astrocytes regulate synapse formation and maturation.

## Visual Abstract

**Figure:**
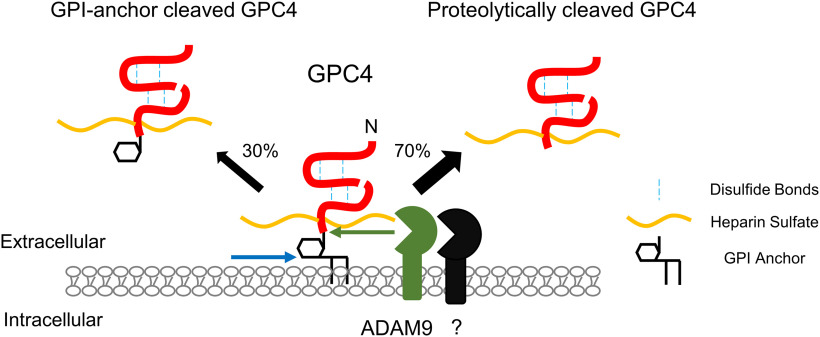

## Significance Statement

Astrocyte-derived diffusible factors regulate synapse development and function. However, the regulatory mechanism underlying the release of astrocyte-derived factors is poorly understood. Noting that, unlike many other secreted factors, glypican-4/GPC4 is GPI-anchored, we characterized the release mechanism of GPI-anchored GPC4 from astrocytes and identified the releasing enzyme. Heparan sulfated GPC4 is robustly released from the astrocytes largely by proteolytic shedding. In particular, ADAM9 in part mediates the release of GPC4 from astrocytes. Our study provides an enzymatic mechanism for releasing GPC4 from astrocytes and will provide a novel opportunity to understand the regulatory mechanism of neuron-glia communication for synaptogenesis.

## Introduction

Surface shedding is a mechanism by which specific surface proteins are cleaved and an ectodomain is released into the extracellular space. The shedding or release of surface proteins have been found to play important roles in various biological functions (Lichtenthaler et al., 2018). Ectodomain shedding can act to stop activity of proteins at the membrane, such as removing cell surface receptors or cell adhesion proteins, as in the case of N-cadherin (Reiss et al., 2005). Alternatively, shedding can release an active protein from the cell surface to act as a long range, diffusible signaling factor such as syndecan-1 in FGF-2 activation during wound healing (Kato et al., 1998). In this context, shedding can allow for temporally and spatially regulated release or activity of a biomolecule. In other cases, shedding acts as a molecular switch, changing the signaling function of the protein from one role while anchored to the surface membrane to another when shed. For example, membrane-anchored TNF-α can activate TNFR2 on direct cell to cell contact to activate anti-inflammatory pathways, however when shed, TNF-α acts as a paracrine signal to drive proinflammatory TNFR1 signaling (Grell et al., 1995). In other cases, released proteins can change the signaling functions of related receptor complexes, such as when released soluble αCNTFR converts the LIF receptor complex and transduces CNTF signaling in the cells that are not normally responsive to CNTF (Davis et al., 1993).

The release of surface proteins also been shown to play important roles in synapse development (Lichtenthaler et al., 2018). For example, Nogo-66 receptor ectodomain shedding drives excitatory synapse formation *in vivo* (Sanz et al., 2018). Proteolytic shedding of cell adhesion molecules causes the structural and functional modifications of synapses (Bajor and Kaczmarek, 2013; Nagappan-Chettiar et al., 2017). Glypican-4/GPC4 has been identified as a cell surface protein sufficient to drive neuronal synaptogenesis *in vitro* (Allen et al., 2012). GPC4 is a GPI-anchored protein (Filmus et al., 2008) and highly expressed in astrocytes (Cahoy et al., 2008; Zhang et al., 2014). Notably, GPC4 expressed in astrocytes are released from the cell and the released GPC4 facilitates synapse maturation *in vitro* (Allen et al., 2012). While GPC4’s downstream mechanism of action of signaling through RPTPs been characterized (Farhy-Tselnicker et al., 2017), little is known about how GPC4 is shed or released from astrocytes. A better understanding of the release mechanism of GPC4 offers insights into the synaptogenic role of astrocytes in neuronal development and could lead to novel therapeutic targets for synaptopathic disorders such as autism, schizophrenia, Down syndrome, and epilepsy (Dowling and Allen, 2018; Wilson and Newell-Litwa, 2018).

GPI-anchored proteins can be released from the producing cells via multiple mechanisms, which include proteolytic shedding, GPI-anchor cleavage, and vesicular release (Muller, 2018). There are GPI-anchored proteins which are shed through proteolytic mechanisms such as metalloprotease-mediated shedding of Prion protein and UL16 binding proteins, MHC Class I-related molecules (Fernández-Messina et al., 2010; Linsenmeier et al., 2018). GPI-anchored proteins can also be shed through a lipase-based cleavage mechanism (Park et al., 2013; van Veen et al., 2017). In this context, unlike proteolytic shedding which releases an ectodomain fragment, an entire protein is released from the cell. GPI-anchored proteins have also been shown to associate with a vesicular membrane or lipid particle while becoming separated from the cell (Muller, 2018).

The mechanism by which GPC4 is released has implications to the role GPC4 plays in downstream signaling. GPC4 is posttranslationally modified with heparan sulfate side chains at residues near the C terminus of the protein. These heparan sulfate side chains are necessary for the known synaptogenic signaling functions of GPC4 (Allen et al., 2012; Condomitti et al., 2018). Thus, identification of the released form of GPC4 is critical to understand how astrocyte-derived GPC4 regulates synaptogenesis.

Here, we show that GPC4 is released from the cell surface of astrocytes *in vitro*. We observed that GPC4 is released predominantly via proteolytic shedding, and to a lesser extent by GPI-anchor cleavage, but not by vesicular release. The released GPC4 contains heparan sulfate attachment, suggesting that both release mechanisms preserve the synaptogenic activity of GPC4 in the extracellular space. We also identify ADAM9 as a proteolytic sheddase for GPC4. Overall, these observations show that astrocytes release heparan sulfate containing GPC4 by multiple mechanisms.

## Materials and Methods

### Animals

All animal care and experiments were conducted in accordance with NIH guidelines and the IACUC committee of the University of Utah (protocol no. 21-02004). C57Bl6/J mouse lines were maintained under the normal housing conditions with food and water available *ad libitum* and 12/12 hour (h) light/dark cycle in a dedicated facility. All primary astrocyte cultures were generated using neonatal wild-type (WT) mice of either sex.

### Cell culture

Heterologous cell experiments were conducted using HEK293T cells. Cells were grown in culture media: DMEM (Invitrogen 11965-092), 10% FBS (Invitrogen 16140071), 1% sodium pyruvate (Invitrogen 11360070), 1% penicillin/streptomycin (Invitrogen 15140-122). For Biochemistry experiments, cells were seeded between 0.15 and 0.2 million cells/well in PEI coated 12-well plates. Plasmid constructs (pCAG nHA GPC4; see Table 1) were transfected with Fugene (Promega E2691)/opti-MEM mixture according to manufacturer protocols; 24 h after transfection, culture media is changed to serum-free DMEM for biochemical experimental conditions.

**Table 1:** Primers used

Set	FP/RP	Primer sequence	Gene name	Vector backbone
Set 1	FP	CGATGGCCGGCCACCATGGCACGCTTAGGCTTGCTC	*Gpc4*	pCAG GPC4
	RP	GCACTTCCGAGCAACTTTTC
Set 2	FP	CCACCATGGCACGCTTAGGCTTGCTCGCGCTCCTCTGCACCCTGGCCGCGCTCAGCGCCTCGCTGCTGGCTGATATCGCGGAGCT	*Gpc4* (endogenous signal peptide)	pCAG GPC4
	RP	CCGCGATATCAGCCAGCAGCGAGGCGCTGAGCGCGGCCAGGGTGCAGAGGAGCGCGAGCAAGCCTAAGCGTGCCATGGTGGCCGG
Set 3	FP	TCGCTGCTGGCTGATATGGTCTTCACACTCGAAGATTTCG	Nano luciferase	pCAG GPC4
	RP	CTTGAGCTCCGCGATCTTATCGTCGTCATCCTTGTAATCCGCCAGAATGCGTTCGCACAG
Set 4	FP	ATAGGCTAGCCTCGAGACCATGGCACGCTTAGGCTTG	Nluc-GPC4	pZac2.1 Nluc-GPC4
	RP	CCGGGTCGACTCTAGATTATCTCCACTCTCCCTGCAC		
Set 4A	RP	ATCGTCTAGATCATTACTTGTACAGCTCGTCCAT	Nluc-GPC4^TM^
Set 5	FP	ATTCTGGCGTCCGGAAAAAAGCGGCCAAAGCCTGGA	Prion	pZac2.1 Nluc-Prion
	RP	TCCAGGCTTTGGCCGCTTTTTTCCGGACGCCAGAAT
Set 6	FP	AATTCGCGCCTAGGTCCGAACCCCGTTCGGACCTAGGCGCGAATTATTTTT	shRNA control	pSilencer 1.0 U6
	RP	AATTAAATTCGCGCCTAGGTCCGAACGGGGTTCGGACCTAGGCGCGAATTGGCC
Set 7	FP	ACAAGAACCACAATTACTAGATCAAGAGTCTAGTAATTGTGGTTCTTGTATTTTT	*Adam9 shRNA1*	pSilencer 1.0 U6
	RP	AATTAAAAATACAAGAACCACAATTACTAGACTCTTGATCTAGTAATTGTGGTTCTTGTGGCC
Set 7A	FP	GCCAATAACGTCTGCATTTTCAAGAGAAATGCAGACGTTATTGGCATTTTT	*Adam9 shRNA2*	pSilencer 1.0 U6
	RP	AATTAAAAATGCCAATAACGTCTGCATTTTCAAGAGAAATGCAGACGTTATTGGCGGCC
Set 8	FP	GCAAATGATACCCTTACAGTTTCAAGAGAACTGTAAGGGTATCATTTGCATTTTT	*Adam10 shRNA1*	pSilencer 1.0 U6
	RP	AATTAAAAATGCAAATGATACCCTTACAGTTCTCTTGAAACTGTAAGGGTATCATTTGCGGCC
Set 8A	FP	GGAAGACAGTTCAACCTACGATCAAGAGTCGTAGGTTGAACTGTCTTCCATTTTT	*Adam10 shRNA2*	pSilencer 1.0 U6
	RP	AATTAAAAATGGAAGACAGTTCAACCTACGACTCTTGATCGTAGGTTGAACTGTCTCCGGCC
Set 9	FP	GGGGTGTGTAACAGCAATAAG	ADAM9	Sequencing primers
	RP	CAAGGGGGACGATTAGGAAG
Set 10	FP	AACAGGCTTATCGCTATG	ADAM10	Sequencing primers
	RP	CTGCTGCTGACTTCTAAT
Set 11	FP	CTGGAGAAACCTGCCAAGTA	GAPDH	Sequencing primers
	RP	AGTGGGAGTTGCTGTTGAAG

**Table 2:** Statistics

Data	Data structure (normality test)	Type of test	Power
Figure 2*D*, lysate measurement of Nluc-GPC4 with and without PI-PLC treatment	Yes	Unpaired *t* test	Cohen’s *d* = 7.29, *p* < 0.0001
Figure 2*D*, media measurement of Nluc-GPC4 with and without PI-PLC treatment	Yes	Unpaired *t* test	Cohen’s *d* = 3.00, *p* < 0.0001
Figure 2*E*, overnight release of GPC4 compared with Prion, normalized to lysate	Yes	Unpaired *t* test	Cohen’s *d* = 3.59, *p* < 0.0001
Figure 3*A*, live surface Biotinylation of GPC4 release over time	Yes	ANOVA; Tukey’s multiple comparisons	Cohen’s *d* = 1.012, *p* < 0.0001
Figure 3*B*, ultracentrifugation of Nluc-GPC4 in ACMs	Yes	Unpaired *t* test	Cohen’s *d* = 7.829, *p* < 0.0001
Figure 3*C*, pulldown of Nluc-GPC4 by α Toxin	Yes	ANOVA; Tukey’s multiple comparisons	Cohen’s *d* = 7.659, *p* < 0.0001
Figure 4*A*, reduction of Nluc-GPC4 by protease inhibitor GM6001	Yes	Unpaired *t* test	Cohen’s *d* = 1.118, *p* = 0.0003
Figure 4*B*, reduction of Nluc-GPC4 release by *Adam9 shRNA1* KD	Yes	ANOVA; Tukey’s multiple comparisons	Cohen’s *d* = 1.832, *p* < 0.0001
Figure 4*B*, reduction of Nluc-GPC4 release by *Adam9 shRNA2* KD	Yes	ANOVA; Tukey’s multiple comparisons	Cohen’s *d* = 2.63, *p* < 0.0001
Figure 4*C*, fold gene expression by qPCR for shRNA KD experiments *Adam9 shRNA1*	Yes	One-sample *t* test	Cohen’s *d* = 1.393, *p* = 0.0190
Figure 4*C*, fold gene expression by qPCR for shRNA KD experiments *Adam9 shRNA2*	Yes	One-sample *t* test	Cohen’s *d* = 0.799, *p* = 0.1076
Figure 4*C*, fold gene expression by qPCR for shRNA KD experiments *Adam10 shRNA1*	Yes	One-sample *t* test	Cohen’s *d* = 10.33, *p* < 0.0001
Figure 4*C*, fold gene expression by qPCR for shRNA KD experiments *Adam10 shRNA2*	Yes	One-sample *t* test	Cohen’s *d* = 1.089, *p* = 0.0444
Figure 4*D*, induction of Nluc-GPC4 release by Adam9 Overexpression	Yes	ANOVA; Tukey’s multiple comparisons	Cohen’s *d* = 3.482, *p* < 0.0001
Figure 5*A*, heparinase treatment of Nluc-GPC4 vs Control	Yes	One-sample *t* test	Cohen’s *d* = 2.93, *p* = 0.0365
Figure 5*B*, pulldown of Nluc-GPC4 by α Toxin in PI-PLC control vs basal release conditions	Yes	Unpaired *t* test	Cohen’s *d* = 0.390, *p* = 0.9467
Extended Data Figure 1-1, cytotoxicity assay of PI-PLC treatment	Yes	ANOVA; Tukey’s multiple comparisons	Cohen’s *d* = 0.907, *p* = 0.964
Extended Data Figure 1-1, cytotoxicity assay of Edelfosine treatment	Yes	ANOVA; Tukey’s multiple comparisons	Cohen’s *d* = 2.870, *p* < 0.0001
Extended Data Figure 2-1, lysate measurement of Nluc-GPC4-TM with and without PI-PLC treatment	Yes	Unpaired *t* test	Cohen’s *d* = 0.49, *p* = 0.19
Extended Data Figure 2-1, media measurement of Nluc-GPC4 = TM with and without PI-PLC treatment	Yes	Unpaired *t* test	Cohen’s *d* = 0.50, *p* = 0.27

Astrocyte culture was generated from P2 mice of either sex. P2 mouse cortexes are dissected and incubated in a Papain solution for 30 min at 37°C. The tissue is then washed with glial media [MEM (Mediatech 15-010-CV), 10% horse serum (HyClone SH30074.03), 1% penicillin/streptomycin/glutamine (Invitrogen 10378-016)] before pipette dissociation and filtering through a 70 μm strainer. Astrocytes are plated on a Poly-D lysine (Millipore A-003-E)/collagen (Advanced BioMatrix 5005-100ML)-coated T75 flask (Fisher 07-202-000). Astrocytes are grown to confluency, with complete media changes every 3–4 d. Once confluent, astrocytes are harvested with an 8 min TrypLE digestion and resuspended in glial media. Astrocyte cell suspension is nucleofected using the Lonza Nucleofector 4D system at 3-μg plasmid DNA per construct (pZac2.1 Nluc-GPC4, pZac2.1 Nluc-GPC4^sec^, pZac2.1 Nluc-GPC4^TM^, pZac2.1 Nluc-Prion, pSilencer 1.0 U6 *shRNA*; see Table 1), pCMV3 C-HA ADAM9 (Sino Biological MG50044-CY), pCS2 cHA3 mADAM10 (Park et al., 2013) with setting CL-133, before being plated on Poly-D lysine/collagen-coated 12-well plates and allowed to grow to confluency for biochemical experiments. Once confluent, glial media is changed to DMEM high-glucose (Invitrogen 11960069) media for biochemical assays and experimental treatments.

### Cloning

GPC4 was cloned from mouse cDNA library from RT-PCR and inserted into a pCAG vector (Table 1, set 1). An HA tag was inserted after GPC4’s signal peptide sequence by long primer (Table 1, set 2). This construct, pCA N-HA GPC4, was used for HEK293T cell experiments. Nluc-GPC4 was developed by insertion of Nanoluciferase (Nluc; from Addgene plasmid #66579) after the cleavage site of the GPC4 signal peptide (Table 1, set 3), then cloning the resulting construct into a pZac2.1 GFABC1D vector (Addgene 92281; Table 1, set 4). A secreted form of GPC4 (Nluc-GPC4^sec^) was generated by introducing a stop codon before the C-terminal GPI-anchorage signal. A transmembrane form of GPC4 (Nluc-GPC4^TM^) was generated by replacing the C-terminal GPI anchorage signal with rat CD2 transmembrane protein (NM_012830.1). Two PCR amplicons including the GPC4 lacking the C-terminal GPI-anchorage signal and CD2 antigen lacking the N-terminal signal peptide were served as the PCR templates to produce chimeric DNA, which is further subcloned into pZac2.1 GFABC1D vector. Nluc-Prion was cloned from RT-PCR of mouse cDNA library and inserted into pZac2.1 GFABC1D vector (Table 1, set 5). *shRNA* constructs were designed *in silico*, and cloned into pSilencer 1.0 U6 with long primers (Table 1, sets 6, 7, and 8 for control, *Adam9*, and *Adam10*, respectively).

### Pharmacology

Astrocytes were cultured as described above. Before treatment, the media of astrocytes were changed to serum-free media and incubated overnight. Astrocytes are then changed into fresh serum-free media with and without treatment (25 μm GM6001) and incubated for 3 h before collection and astrocytes are lysed for biochemical analysis or luciferase assay.

Phosphoinositide phospholipase C (PI-PLC) is a bacterial enzyme that robustly cleaves GPI-anchored proteins from the cell surface membrane. For biochemical assays, PI-PLC is added to cell culture media for 0.5–3 h for acute treatment or overnight for chronic treatment. Both time frames result in near complete release of all GPI-anchored proteins from the cell surface.

### Toxicity assay

Cell death in primary astrocyte cultures was assayed using the Lonza ToxiLight Cytotoxicity BioAssay kit (Lonza LT17-217). Briefly, 20 μl of cultured media was collected, and mixed with 100-μl kit detection reagent. Samples were incubated for 5 min, then luminescence was measured with a plate reader. Higher levels of luminescence are driven by the release of adenylate kinase from damaged cells, which react with ADP and luciferase in the assay buffer to produce bioluminescence. A toxic concentration of Edelfosine (50 μm) was used as a positive control for cell death.

### Western botting

Media samples were mixed with 4× gel loading buffer (200 mm Tris, 8% SDS, 24% glycerol, and 0.04% bromophenol blue, pH 6.8), while lysate samples were created by lysing cells with 1× gel loading buffer and syringe lysing. Where necessary, astrocyte conditioned media (ACMs) samples were concentrated 20× in 10 kDa cutoff centrifuge protein concentrators (Thermo Fisher Scientific 88517) to enrich for WT GPC4 before mixing with gel loading buffer. Samples were loaded into 10% gels and run at 140 V for 70 min, then transferred at 100 V for 60 min on PVDF membranes. Membranes were blocked for 1 h with 5% milk in TBST (100 mm Tris, 150 mm NaCl, and 0.1% Tween 20, pH 7.5) before placed in primary antibody: HA (BioLegend 901501), GAPDH (Cell Signaling Technology 2118S), IGFBP2 (Cell Signaling Technology 3922S), Nluc (R&D Biosystems MAB100261), and CD9 (Abcam ab92726). Blots were probed with HRP-conjugated secondaries [mouse (Jackson ImmunoResearch 715-035-151), rabbit (Jackson ImmunoResearch 705-035-003), rat (Jackson ImmunoResearch 112-035-003)]. Blots were developed using a chemiluminescent substrate (Thermo Scientific 34580).

### Luciferase assay

Promega Nanoluc Furimazine is diluted 1:50 in assay substrate:assay buffer, then diluted 1:4 in 1× PBS. Media samples are cleared by centrifugation and 20 μl of sample are loaded in 96-well plate wells. Lysate samples are generated by treatment of 1× passive lysis buffer (Promega E1941) to 12-well plate cultures, and 20 μl of sample are loaded in 96-well plate wells. Luminescence of individual wells was read in a plate reader after injection of 100 μl Furimazine. Average luminescence value is calculated and normalized to either pretreatment or lysate samples as a transfection control.

### Biotinylation

Cells are cooled on ice, then washed with PBS ++ (PBS, 1 mm CaCl_2_, and 0.5 mm MgCl_2_, pH 7.4) before 10-min incubation of Sulfo-NHS-SS-biotin (Invitrogen 21331; 1 mg/ml in PBS++). Biotin washed off with PBS++, then quenched with cold glial media, then washed again in PBS before incubated in serum-free media for 5 h at 37°C. Incubation media collected, then cleared by centrifugation; 300 μl 1:1 slurry strepavidin beads (Invitrogen 29201) were prepared with a 1× RIPA (150 mm NaCl, 1% P-40, 0.5% NaDOC, 0.1% SDS, and 25 mm Tris) wash, two wash buffer rinses (40 mm Tris, pH 7.6) before resuspended in wash buffer as 1:1 slurry; 40 μl 1:1 slurry used per 900 μl media sample and incubated together with rotation at 4°C for 1 h. Beads are spun down, supernatant is collected for analysis, then beads are washed with 1× wash buffer. Bead fraction is resuspended in elution buffer (2 m NaCl and 40 mm Tris, pH 8) and slurry is used for Western blot and Nluc assays.

### Ultracentrifugation

Astrocytes are grown after nucleofection in T75 flasks until confluent. Astrocytes are incubated in serum-free media for 24 h before media is collected and subjected to serial centrifugations (10 min 300 × *g*, 10 min 2000 × *g*, 30 min 10,000 × *g*). A 1.5 h, 100,000 × *g* centrifugation pellets extra cellular vesicles (Konoshenko et al., 2018). The supernatant and pellet are collected for Nluc assays and Western blot analysis.

### Toxin pulldown

α-Toxin (AT) plasmid was generously provided by Yeongjin Hong at the Chonnam National University, Korea (Shin et al., 2006). BL21 cells (Fisher NC9122855) expressing α-toxin are pelleted and resuspended in 0.5× RIPA (75 mm NaCl, 0.5% P-40, 0.25% NaDOC, 0.05% SDS, and 12.5 mm Tris) before being sonicated. Cell extract is filtered and incubated with washed TALON beads (Takara 635501) at 4°C for 30 min. After washing, AT-coated TALON beads are resuspended as 1:1 slurry in wash buffer (20 mm Tris, 500 mm NaCl, pH7.9), and 60 μl of slurry is added to 900 μl of media samples and incubated for 1 h at 25°C. Beads are pelleted by centrifugation, supernatant is collected, beads are washed with wash buffer before being resuspended as a 1:1 slurry in wash buffer and used in Nluc assays.

### DEAE pulldown

DEAE-Sepharose beads (Sigma DFF100-50ML) are prepared with sequential high and low salt washes (2 m NaCl, 40 mm Tris, pH 8.0, and 40 mm, Tris pH 7.6, respectively) before resuspended as a 1:1 slurry in wash buffer. Media containing Nluc-GPC4 was added to DEAE 1:1 slurry, and incubated at room temperature with rotation for 2 h. Media-DEAE mixture is centrifuged and supernatant is collected while the DEAE pellet is washed once with 10× bed volume wash buffer before wash buffer is aspirated. Nluc-GPC4 is eluted from DEAE beads with high salt (40 mm Tris and 2 m NaCl, pH 8) at room temperature for 30 min with mild agitation. Eluted Nluc-GPC4 in elution buffer is collected and used for luciferase assay.

### Heparinase treatment

Samples containing Nluc-GPC4 were treated with Heparinase II (NEB P0735S) and Heparinase III (NEB P0737S) overnight at 37°C, then subject to DEAE pulldown described above, before luciferase activity was assayed. Untreated samples were similarly incubated at 37°C and used as controls for Heparinase treatment for the DEAE pulldown and luciferase assay.

### Quantitative PCR (qPCR)

Astrocytes were harvested 4 d after *shRNA* nucleofection and subjected to RNA extraction using TRI Reagent Solution (Invitrogen, AM9738). 1 μg of extracted RNA was used for the RT reaction using PrimeScript High Fidelity RT-PCR kit (Takara, R022A), according to the manufacturer`s instruction. 0.5 μl from the total 20 μl cDNA eluent was mixed with each 0.22 μm *Adam9* primers (Table 1, set 9), *Adam10* primers (Table 1, set 10), or *Gapdh* (Table 1, set 11) with 23 μl SYBR Select Master Mix (Applied Biosystems, 4472908). The PCR was conducted with total 40 cycles (20 s at 95°C, 20 s at 58°C, and 40 s at 72°C), using a Life Technologies QuantStudio 12K Flex instrument at the Genomics Core Facility, a part of the Health Sciences Cores of the University of Utah. *Adam* mRNA levels in the *shRNA* nucleofection was normalized by that of *Gapdh* and was compared with *shRNA* control nucleofection conditions.

### Statistical analyses

Statistics were performed using GraphPad Prism software version 9 (Table 2). Experiments with two groups were compared with unpaired *t* tests and 95% confidence intervals (CIs) were calculated for group means and the difference between means. Experiments with three or more groups were compared with ANOVA and Tukey’s multiple comparisons, and 95% CIs were calculated for group means and the differences between means. One-sample *t* tests were used for Figures 4*C*, 5*A* to calculate the difference between control and experimental groups. All data are plotted as mean, with error bars denoting 95% CIs and all individual data points plotted. For all experiments, the *n* numbers shown refer to the number of replicates, while *N* numbers refer to the number of biological replicates.

## Results

### GPC4 is expressed on the astrocyte surface via a GPI-anchor.

**Figure 1.**
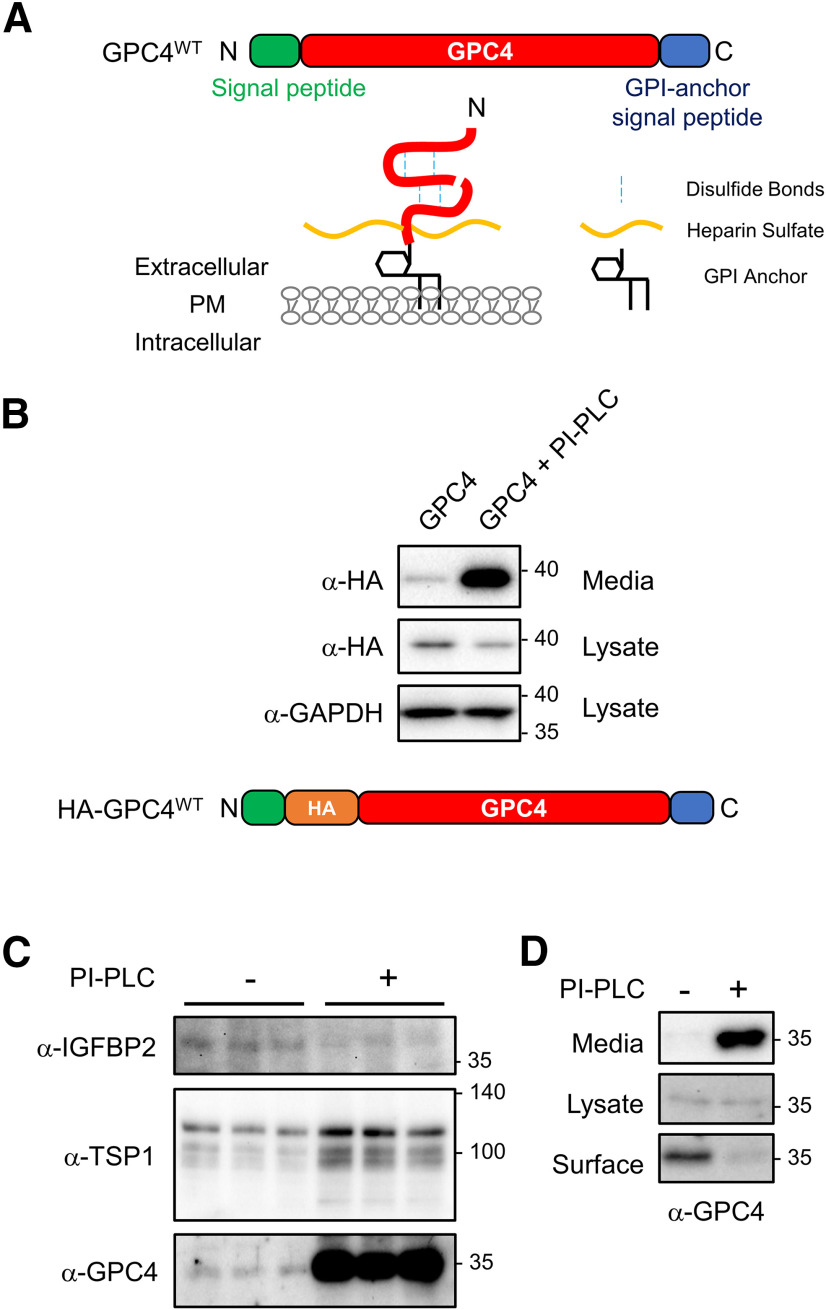
GPC4 is expressed on the astrocyte surface via a GPI-anchorage. ***A***, Diagram of GPC4 sequence, protein, and posttranslational modifications. GPC4 contains an N-terminal signal peptide and GPI-anchor signal peptide for GPI-anchor attachment. Mature GPC4 is subject to furin cleavage, intrachain di-sulfide bonds, heparan sulfate attachment, and GPI-anchorage to the cell surface membrane. ***B***, Western blotting of HA tagged GPC4 construct expressed in HEK293T cells. The ∼37-kDa band shows the HA tagged N-terminal GPC4 in the reducing condition. PI-PLC treatment in HEK293T cells drives robust release of GPC4; however, GPC4 is detected in media in the absence of PI-PLC. ***C***, Western blotting of concentrated ACMs for endogenous GPC4, IGFBP2, and TSP1, with and without PI-PLC treatment. PI-PLC treatment strongly facilitates release of endogenous GPC4 from astrocytes. ***D***, Biotinylation of surface GPC4 shows that pretreatment of PI-PLC removes endogenous GPC4 from the cell surface of astrocytes. Extended Data Figure 1-1 supports Figure 1.

*In silico* analysis indicates that GPC4 contains the N-terminal signal peptide and C-terminal hydrophobic patch and is predicted to be a GPI-anchored protein (Fig. 1*A*). To directly test whether GPC4 is GPI-anchored on the cell surface, we first examined whether GPC4 is released from the cell surface by the application of PI-PLC, a bacterial GPI-anchor cleaving enzyme (Chen et al., 1998). N-terminal HA-tagged GPC4 was transfected into HEK293T cells, and media were collected with and without PI-PLC treatment (Fig. 1*B*). PI-PLC treatment greatly facilitated the release of GPC4, indicating that GPC4 is GPI-anchored on the HEK293T cell surface. Interestingly, GPC4 was detected in the medium by Western botting without PI-PLC treatment, suggesting constitutive release of GPC4 from the HEK293T cells.

10.1523/ENEURO.0069-21.2021.f1-1 Extended Data Figure 1-1 Extended data supporting Figure 1*C*. Cell toxicity assay of WT astrocytes with and without overnight PI-PLC treatment. Higher levels of bioluminescence indicate a loss of cell integrity and leakage of intracellular adenylate kinase into the media. Edelfosine treatment (50 μm, 1 h) is included as a positive control for cell death. Tukey’s multiple comparison Ctrl versus PI-PLC *p* = 0.964, Cohen’s *d* = 0.907. Tukey’s multiple comparison Ctrl versus Edelfosine *p* < 0.0001, Cohen’s *d* = 2.870. Download Figure 1-1, TIF file.

Next, we tested whether endogenous GPC4 expressed on the astrocytes is GPI-anchored. Primary astrocytes from mouse cortex were treated with or without PI-PLC overnight and collected ACMs was assayed for shed GPC4. Because of the poor antigenicity of GPC4, we concentrated the ACM for Western bottings (see Materials and Methods) Consistent with observations in heterologous cells, PI-PLC treatment on astrocytes greatly facilitated the shedding of endogenous GPC4 into the media (Fig. 1*C*). In contrast, PI-PLC treatment did not increase the secretion of IGFBP2 from astrocytes. Interestingly, PI-PLC treatment also slightly increased the level of thrombospondin 1 (TSP1) in the culture medium, an astrocyte secreted protein involved in synaptogenesis (Christopherson et al., 2005). This result suggests that a portion of secreted TSP1 is associated with GPI-anchored proteins on the astrocyte surface. TSP1 is known to bind surface heparan sulfate, and a portion of secreted TSP1 from astrocytes may be recruited on the astrocyte surface, possibly as a mechanism to control the diffusion of secreted TSP1 (Feitsma et al., 2000). To further test whether PI-PLC treatment non-specifically releases cellular contents from astrocytes, we performed a cell toxicity assay. Overnight treatment of astrocytes with PI-PLC did not affect the cell viability, while edelfosine treatment (50 μm, 1 h), which induces apoptosis by activating Fas/CD95 receptor and PLC inhibition (Mollinedo et al., 2004), caused astrocyte cell death (Extended Data Fig. 1-1).

To determine the degree of GPI-anchorage of GPC4 on the astrocyte cell surface, we performed a surface biotinylation assay, which allows for labeling of all surface proteins at the time of biotinylation. PI-PLC pretreatment removed the biotinylated GPC4 from the cell lysate (Fig. 1*D*). These results indicate that GPC4 is expressed on the astrocyte surface predominantly via GPI-anchor.

### The release of GPC4 from astrocytes

**Figure 2.**
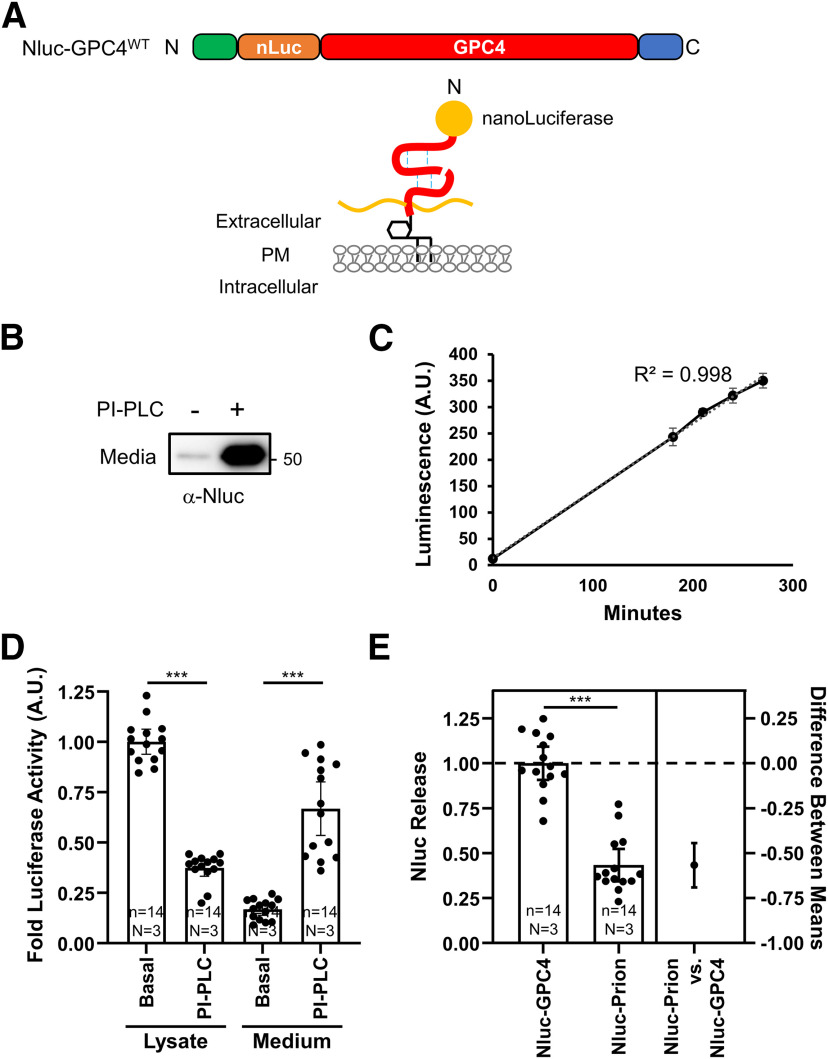
Luciferase assay for quantifying the release of GPC4 from astrocytes. ***A***, Nluc is inserted at the N terminus, after the endogenous signal peptide, to preserve GPC4 trafficking. ***B***, Primary astrocytes were nucleofected with Nluc-GPC4 and treated with and without PI-PLC. Western botting with α-Nluc antibody showed the expected 20 kDa size shift in the N-terminal fragment. PI-PLC treatment facilitates the release of Nluc-GPC4, confirming the GPI-anchorage of the construct. ***C***, Representative trace of one experiment showing the linear kinetics of GPC4 release from astrocyte culture (*R*^2^ = 0.998). Error bars indicate the standard error of the mean. ***D***, Astrocytes expressing Nluc-GPC4 were incubated in fresh media with and without PI-PLC for 3 h, and Nluc signal was measured in the cell lysate and media. Nluc signal was normalized to untreated lysate conditions for each biological replicate. PI-PLC treatment resulted in the decrease in the luciferase activity of the cell lysate and the corresponding increase in the activity in the media. These data show Nluc-GPC4 is quantitative in measuring released versus surface pools of GPC4. Error bars indicate 95% CI of the mean here and in following graphs. The requirement of the GPI-anchorage for PI-PLC-dependent release of GPC4 is shown in Extended Data Figure 2-1. ***E***, The release rate (media over lysate activity) of Nluc-GPC4 and Nluc-Prion was normalized to Nluc-GPC4 release. Nluc-GPC4 is released ∼2-fold more than Nluc-Prion (*t* test *p* < 0.0001, Cohen’s *d* = 3.59); ***p < 0.001.

Proteomic analysis of ACM showed that GPC4 is highly enriched in the astrocyte secretome, ranking 20th in Greco and colleagues, and ranking 28th in our mass spec analysis (data not shown; Greco et al., 2010). However, GPC4 undergoes extensive posttranslational modifications, including GPI-anchorage, convertase-mediated cleavage, disulfide-linkage, glycosylation, and heparan sulfate attachment (Fig. 1*A*; Filmus et al., 2008), which may cause the poor antigenicity. To quantify release kinetics of GPC4 from astrocytes, we generated a Nluc fusion protein construct after the endogenous signal peptide of GPC4 (Nluc-GPC4; Fig. 2*A*). Nluc is a small (20 kDa) and bright luciferase reacting with a specific substrate, Furimazine (FMZ; Heise et al., 2013). Attaching the signal peptide to the N terminus of Nluc efficiently delivers the fusion protein into the secretory pathway. The secreted Nluc shows a linear luminescence in a wide range of Nluc concentration in the medium, which is suitable for quantifying the release of the membrane-tethered protein.

10.1523/ENEURO.0069-21.2021.f2-1 Extended Data Figure 2-1 Extended data supporting Figure 2*D*. Astrocytes expressing Nluc-GPC4-TM was incubated in fresh media with and without PI-PLC for 3 h, and Nluc signal was measured in the cell lysate and media. Nluc signal was normalized to untreated lysate conditions for each biological replicate. PI-PLC treatment did not result in the release of GPC4-TM from astrocytes. Lysate +/– PI-PLC unpaired *t* test *p* = 0.19, Cohen’s *d* = 0.49. Media +/– PI-PLC unpaired *t* test *p* = 0.27, Cohen’s *d* = 0.50. Download Figure 2-1, TIF file.

We examined the processing, trafficking, and kinetics of the construct as well as the posttranslational modifications important for GPC4 function. We nucleofected the Nluc-GPC4 construct under an astrocyte-specific GFABC1D promoter into astrocytes and measured the release of Nluc-GPC4 into the medium. Western botting with Nluc antibody showed the expected ∼20 kDa shift of N-terminal fragment of GPC4 (Fig. 2*B*). Further, the treatment of PI-PLC robustly releases Nluc-GPC4 into the medium, indicating that N-terminal fusion of Nluc does not affect the GPI anchorage and surface expression of GPC4. It is also possible that PI-PLC treatment indirectly regulates the release of other types of proteins. For example, release of RECK, a GPI-anchored metalloprotease inhibitor from the cell surface can de-repress the surface sheddase, which in turn facilitates the shedding of non-GPI anchored proteins (Park et al., 2013). To test whether the release of Nluc-GPC4 by PI-PLC requires the GPI-anchorage, we generated a transmembrane form of GPC4 by replacing the C-terminal GPI-anchorage signal with a rat CD2 transmembrane protein (Nluc-GPC4^TM^). PI-PLC treatment did not affect the release of Nluc-GPC4^TM^ in astrocytes (Extended Data Fig. 2-1). This result further supports that PI-PLC treatment does not cause toxicity nor non-specific release of cellular proteins. Interestingly we observed linear accumulation of Nluc-GPC4 signal into the medium at the naive state (*R*^2^ = 0.998), further demonstrating the quantitative nature of the Nluc-GPC4 (Fig. 2*C*). Next, we measured the release rate of GPC4 per surface-tethered pool of GPC4. The amount of the surface pool of GPC4 is calculated by the reduction of Nluc-GPC4 signal in the cell lysate and the corresponding increase in the medium signal on PI-PLC treatment (Fig. 2*D*). We observed that 27% of the surface GPC4 is released into the medium over 3 h, which indicates that ∼9% of the surface-expressed pool is released per hour at the naive state.

Noting the high release rate of GPC4, we compared the release rate of Prion, another GPI-anchored protein abundantly expressed in astrocytes (Cahoy et al., 2008; Zhang et al., 2014). The release rate (luminescence of the medium normalized to that of the cell lysate) of Nluc-GPC4 is >2-fold higher than that of Nluc-Prion, suggesting that even in this overexpression system, specific release mechanisms exist for different GPI anchored proteins and feature differential release kinetics (mean difference 95% CI = −0.7588 to −0.4107, Cohen’s *d* = 3.92, *p* < 0.0001; Fig. 2*E*). Overall, Nluc-GPC4 retains posttranslational modifications of endogenous GPC4, including furin cleavage, and GPI-anchorage, and allows for rapid, non-destructive measurement of protein release from the cell surface into the media.

### GPC4 is released from the astrocyte cell surface

How is GPI-anchored GPC4 is released from astrocytes? GPI-anchored proteins can be released from the cell via multiple mechanisms, which include (1) secretory pathway bypassing surface expression because of cleavage during processing in the secretory pathway, e.g., by GPLD1 (Hettmann et al., 2003; Park et al., 2013); (2) vesicular releases such as in exosomes or lipid associated particles (Muller, 2018; Dobrowolski et al., 2020); (3) GPI-anchor cleavage on the cell surface (Park et al., 2013; van Veen et al., 2017); and (4) proteolytic shedding. Thus, we tested each possibility.

**Figure 3.**
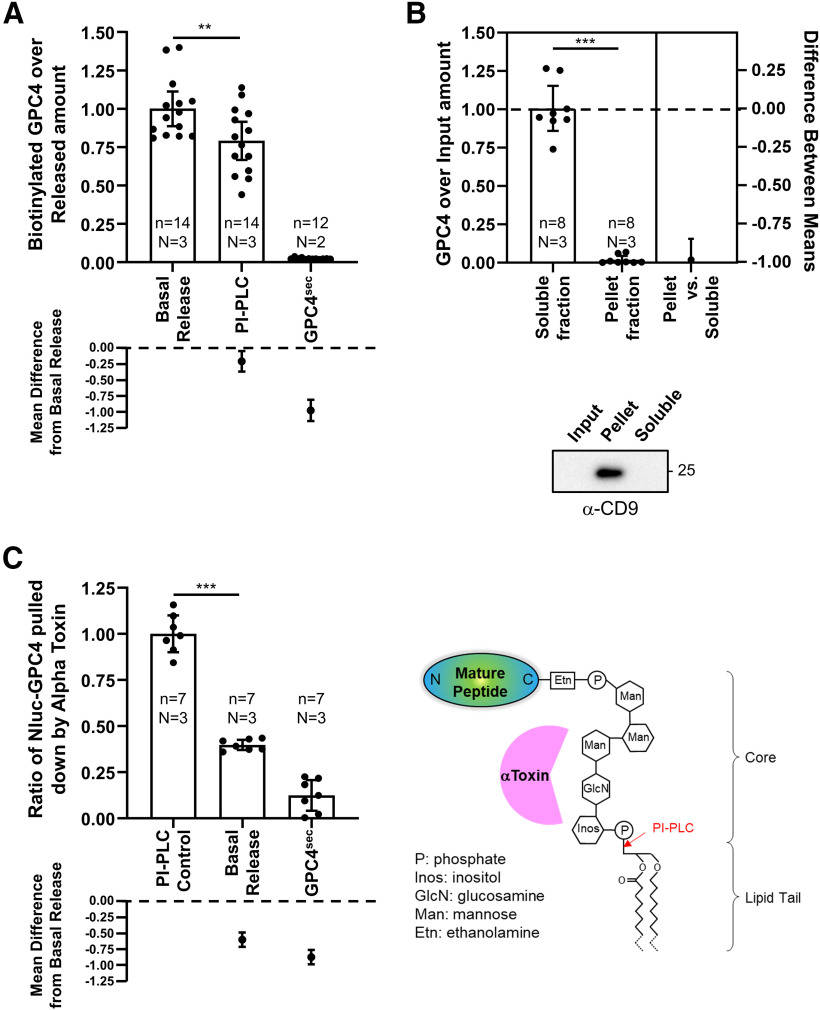
Characterizing the release mechanism of GPC4 using Nluc-GPC4. ***A***, After biotinylating the surface proteins of astrocytes expressing Nluc-GPC4, cells were further incubated in the fresh medium for 5 h. The released Nluc-GPC4 is collected from the medium, and the ratio of biotinylation of the released Nluc-GPC4 was measured (luciferase activity of avidin pulldown over medium input). As a control for the release from the cell surface, PI-PLC were treated during the incubation time. Both basal and PI-PLC induced GPC4 release occur from the cell surface. GPC4^sec^, which does not contain the GPI-anchor signal peptide and is thus not GPI-anchored, is directly secreted without membrane attachment and lacks biotinylation (basal vs PI-PLC ANOVA *p* = 0.0079, Cohen’s *d* = 1.012). ***B***, Ultracentrifugation of ACMs containing Nluc-GPC4 was used to test for GPC4 association with extracellular vesicles as a potential release mechanism. Supernatant (soluble) fraction and pellet (vesicular) fraction Nluc were normalized to input Nluc luminescence. Nluc-GPC4 did not associate with the pellet (vesicular) fraction. An exosome marker, CD9, was enriched in the pellet (vesicular) fraction (*t* test *p* < 0.0001, Cohen’s *d* = 7.829). ***C***, alpha toxin (AT), which binds to the glycan core of GPI-anchors, was used to pull down GPI-anchored proteins released through lipase activity. PI-PLC, lipase released Nluc-GPC4 is used as a positive control, while GPC4^sec^, lacking a GPI anchor, is used as a negative control. Nluc-GPC4 pulled down by AT is normalized to input Nluc-GPC4 measurements. Basal release shows partial binding to AT, indicating that ∼30% of Nluc-GPC4 is released through a lipase mechanism (PI-PLC vs basal release ANOVA *p* < 0.0001, Cohen’s *d* = 7.659); **p < 0.01, ***p < 0.001.

We first tested whether extracellular GPC4 at the basal state is shed from the cell surface or directly secreted without being retained on the astrocyte surface membrane. To test this possibility, we conducted a live surface biotinylation assay wherein astrocytes cell surface proteins were biotinylated before being allowed to recover and incubate at 37°C for 5 h. Incubation media was then collected and assayed for biotinylated GPC4. If GPC4 is secreted directly from secretory pathways, the majority of collected GPC4 should not be biotinylated. Instead, we found that the ratio of biotinylated over total released GPC4 is similar between the basal state and the PI-PLC condition, which selectively releases the surface-bound pool (Fig. 3*A*). In contrast, a secreted form of Nluc-GPC4 (GPC4^sec^), which lacks the C-terminal GPI-anchorage signal, is secreted without being biotinylated. While we expected the PI-PLC treatment condition to result in a maximal biotinylation ratio, the biotinylation ratio of the basal release was slightly higher than that of PI-PLC conditions (mean difference between basal vs PI-PLC 95% CI = −0.3189 to −0.1594, Cohen’s *d* = 1.012, *p* = 0.0079). PI-PLC treatment rapidly removes all surface GPC4, which may facilitate the surface incorporation of newly synthesized, un-biotinylated GPC4. This would increase the release of un-biotinylated GPC4 by PI-PLC during the 5-h incubation as compared with untreated control. Together, these data demonstrate that GPC4 is released from the astrocyte surface membrane and not directly from a secretory pathway (Fig. 3*A*).

### GPC4 is not released through an extracellular vesicle mechanism

Several GPI-anchored proteins are enriched in the extracellular vesicles (Vidal, 2020). These vesicular GPI-anchored proteins retain the lipid tail and are associated with membrane structure. We tested for the possibility that GPC4 is released through an extra vesicular fraction like some GPI anchored proteins such as CNTFR (Choi et al., 2021). In this model of release, GPI-anchored GPC4 is expressed on the cell surface, and released while still bound to the membranes of extracellular vesicles either through exosomal or ectosomal pathways. To test this, media from astrocytes expressing Nluc-GPC4 were sequentially centrifuged, first to separate large particles such as floating cells and cell debris, then to isolate the vesicular fraction by ultracentrifugation (Konoshenko et al., 2018). We measured luciferase activity in the supernatant and pellet fractions, which correspond to soluble protein and vesicle associated fractions, respectively, and normalized the measurements to luciferase activity in the input sample. Luciferase activity shows no change between input and supernatant fractions, with no detection in the vesicular pellet fraction. To validate the ultracentrifugation assay, we tested the pellet fraction and confirmed enrichment of exosomal protein CD9 (Fig. 3*B*). Together, these data show minimal GPC4 presence in vesicular fractions, suggesting that extracellular vesicles are not the major route of GPC4 release in astrocytes.

### GPC4 is partially released through a GPI-anchor lipase

A GPI-anchor lipase such as GDE family enzymes and GPLD1, cleaves the GPI anchor via PLC and PLD mechanism, respectively, and leaves the sugar core attached to the C terminus of the released protein. In contrast, a proteolytic cleavage would release a GPC4 ectodomain fragment that lacks the GPI-anchor. Noting that AT, a bacterial GPI-anchor binding protein, recognizes the sugar motif of the GPI-anchor (Hong et al., 2002; Shin et al., 2006), we developed a pulldown assay that isolates the species containing the core domain of the GPI-anchor among the released pool (Fig. 3*C*). We expressed AT-His in *Escherichia coli* and purified it with metal resins. To test between these possibilities, we first assayed the percentage of released GPC4 with GPI-anchor residues using a pulldown experiment. Surprisingly, we found that in comparison to our positive PI-PLC control, which releases the surface GPC4 through GPI-anchor lipase activity, constitutively released GPC4 is only partially captured by AT-coated beads (mean difference between PI-PLC control vs basal release 95% CI = −0.4897 to −0.7146, Cohen’s *d* = 7.659, *p* < 0.0001; Fig. 3*C*). This suggests that while some GPC4 is released through lipase activity of the GPI anchor, a majority of GPC4 is released through a mechanism that removes the GPI anchor from the released form.

### GPC4 is partially released through a proteolytic mechanism

**Figure 4.**
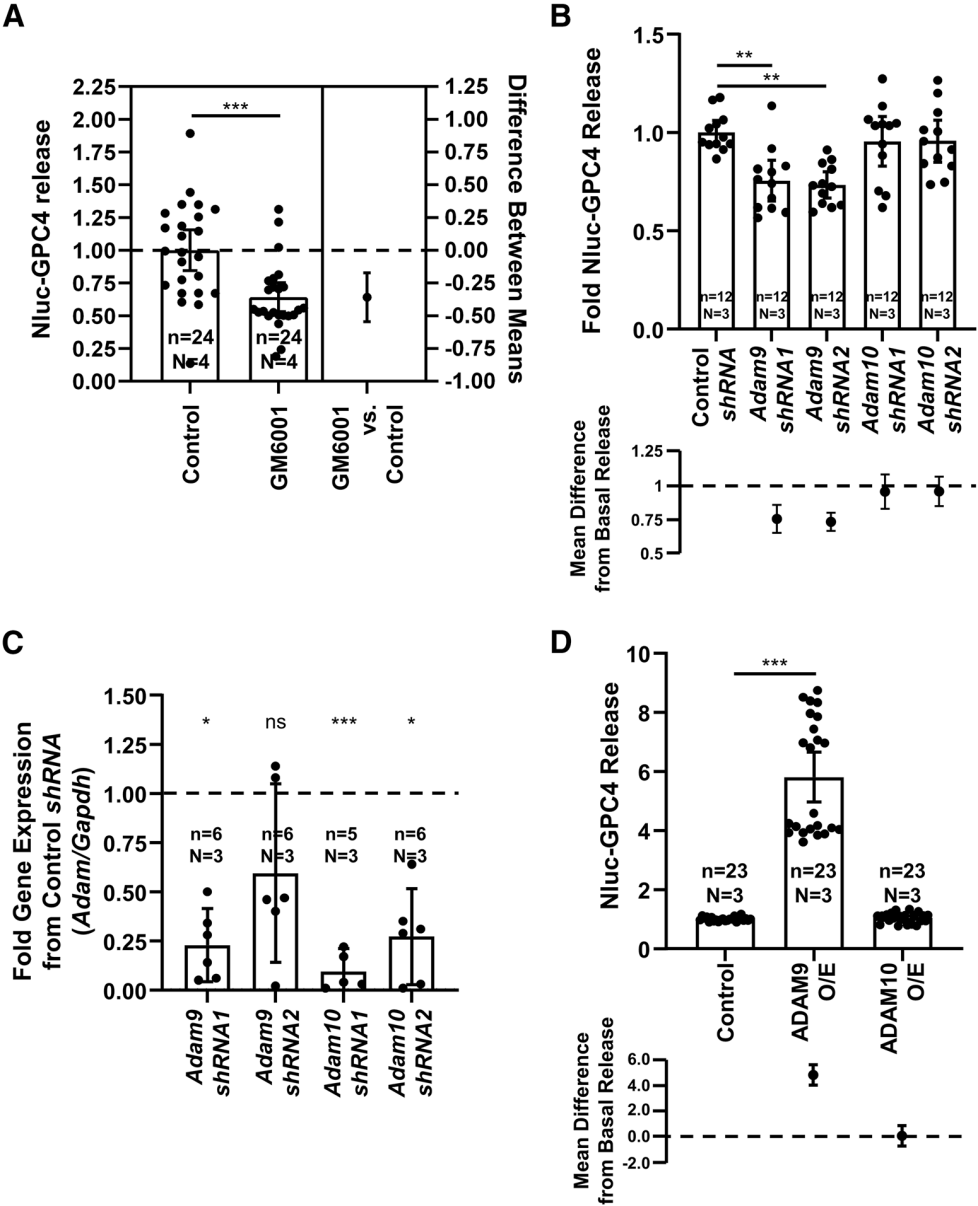
ADAM9 mediates the release of GPC4 from astrocytes. ***A***, GM6001 (25 μm), an inhibitor of Zn^2+^-based proteases including the MMP and ADAM family proteases, reduced the release of GPC4 (*t* test *p* = 0.0003, Cohen’s *d* = 1.118). ***B***, *shRNA* knock-down of *Adam9* and not *Adam10* reduces the release of Nluc-GPC4 in astrocytes (control vs *Adam9 shRNA1* Tukey’s *p* = 0.0019, Cohen’s *d* = 1.832; control vs *Adam9 shRNA2* Tukey’s *p* = 0.0010, Cohen’s *d* = 2.63; control vs *Adam10 shRNA1* Tukey’s *p* = 0.8999, Cohen’s *d* = 0.291; control vs *Adam9 shRNA2* Tukey’s *p* = 0.8999, Cohen’s *d* = 0.315). ***C***, qPCR data of astrocyte *Adam9* and *Adam10* knock-down replicates. Δ-CT between *Adam* and *Gapdh* and ΔΔ-CT between control and target *shRNA* conditions were used to determine the fold change in expression. One biological replicate for *Adam9 shRNA2* did not show reductions in *Adam9* gene expression. *Adam9 shRNA1* one-sample *t* test *p* = 0.0190, Cohen’s *d* = 1.393; *Adam9 shRNA2 p* = 0.1076, Cohen’s *d* = 0.799; *Adam10 shRNA1 p* < 0.0001, Cohen’s *d* = 10.338; *Adam10 shRNA2 p* = 0.0444, Cohen’s *d* = 1.089. ***D***, ADAM9 overexpression, and not ADAM10 overexpression (O/E), is capable of inducing the release of Nluc-GPC4 from astrocytes (control vs ADAM9 O/E ANOVA *p* < 0.0001, Cohen’s *d* = 3.482); *p < 0.05, **p < 0.01, ***p < 0.001, ns: not significant.

The results of the AT pulldown immediately suggest proteolytic cleavage as the primary mechanism by which GPC4 is released from the astrocyte cell surface. We first examined the effect of protease inhibitors on GPC4 release. In line with our model of proteolytic cleavage, we observed ∼30% reduction in GPC4 release after treatment with a metalloprotease inhibitor GM6001, an inhibitor of Zn catalyzed proteases, including MMP and ADAM family proteases (mean difference 95% CI = −0.5445 to −0.1723, Cohen’s *d* = 1.118, *p* = 0.0003; Fig. 4*A*; Webber et al., 2002).

### Determining the protease responsible for GPC4 shedding

GM6001 treatment significantly blocked the basal release of GPC4, indicating that Zn-dependent MMPs and ADAM family proteases play a role in GPC4 shedding (Fig. 4*A*). To identify the protease that mediates GPC4 cleavage, we performed a gain of function screen with tissue inhibitors of metalloproteinases (TIMPs) which inhibit MMP and ADAM proteases (Brew and Nagase, 2010). Overexpression of TIMP 1 and TIMP 3, which collectively inhibit MMPs and ADAMs 10, 12, 17, 28, and 33, was unable to reduce release of GPC4 from astrocytes (data not shown). These results suggest that GPC4 shedding may be mediated by other proteases that are not inhibited by TIMPs, which include ADAMs 8, 9, and 19 (Brew and Nagase, 2010).

In parallel, we performed *shRNA* screens of ADAM proteases (*Adamts1*, *Adamts5*, *Adam9*, *Adam10*, *Adam17*) that are abundantly expressed in astrocytes (data not shown; Li et al., 2019). Among ADAM family proteases we tested, we observed that *shRNAs* against *Adam9* significantly reduced the release of GPC4 (*Adam9 shRNA1* mean difference 95% CI = −0.34 to −0.14, Cohen’s *d* = 1.832, Tukey’s *p* = 0.0019; *Adam9 shRNA2* mean difference 95% CI = −0.33 to −0.19, Cohen’s *d* = 2.68, Tukey’s *p* = 0.0010; Fig. 4*B*). Consistent with our observations of TIMP overexpression, *Adam10 shRNA* produced little effect on the release of GPC4 from astrocytes (*Adam10 shRNA1* mean difference 95% CI = 0.172 to −0.080, Cohen’s *d* = 0.291, Tukey’s *p* = 0.8999; *Adam10 shRNA2* mean difference 95% CI = 0.150 to −0.063, Cohen’s *d* = 0.315, Tukey’s *p* = 0.8999; Fig. 4*B*).

qRT-PCR of wells transfected with a *shRNA* construct showed knock-down efficiency ranging from 0.1- to 0.5-fold except in one biological replication of *Adam9 shRNA2* (Fig. 4*C*). Incomplete knock-down may cause mild effect of *Adam9* knock-down on GPC4 release. Interestingly, reductions in Nluc-GPC4 release are consistently seen in *Adam9 shRNAs* and not by *Adam10 shRNAs*. Since double nucleofection rate is near complete within the transfected cells and the release rate of Nluc-GPC4 is insensitive to the transfection rate after normalizing the luminescence in the lysate (data not shown), knock-down of *Adam9* within the same cells expressing Nluc-GPC4 may produce more consistent effects on Nluc-GPC4 release than overall reduction of the mRNA level of the culture. Taken together, these results indicate that ADAM9 in part mediates the release of Nluc-GPC4.

To test whether ADAM9 is sufficient to induce the release of GPC4 from astrocytes, we overexpressed ADAM9 together with the Nluc-GPC4 reporter. Our results show that overexpression of ADAM9 and not ADAM10 facilitated GPC4 release from astrocytes (ADAM9 mean difference 95% CI = −5.606 to −4.007, Cohen’s *d* = 3.482, *p* < 0.0001; ADAM10 mean difference 95% CI = −0.8448–0.7549, Cohen’s *d* = 0.1542, *p* = 0.99; Fig. 4*D*). Taken together, these data show that ADAM9 or its downstream effectors facilitate the release of GPC4 from astrocytes.

### Constitutively shed GPC4 predominantly contains the heparan sulfate side chains

**Figure 5.**
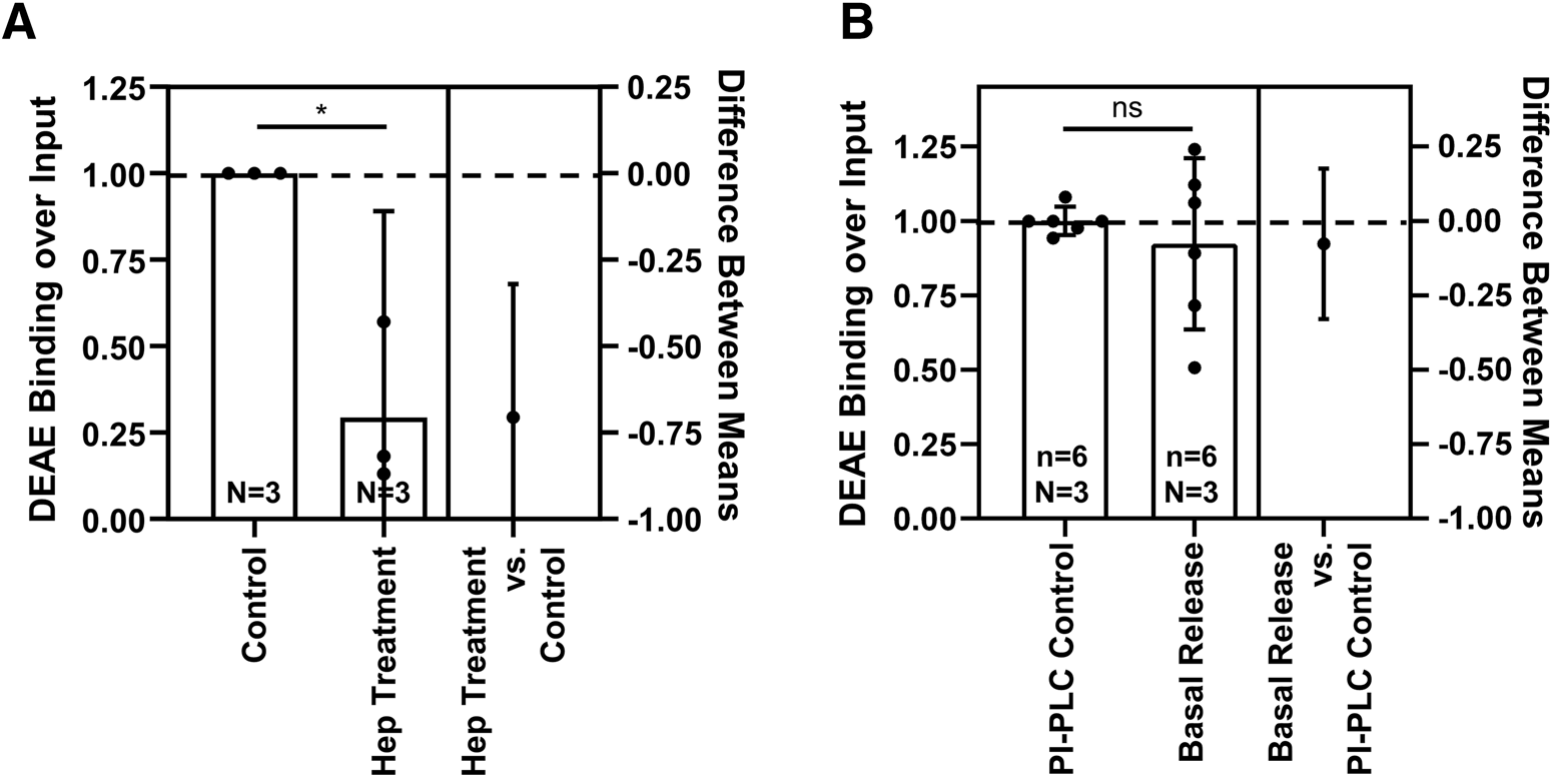
The released Nluc-GPC4 includes the heparan sulfate side chain. ***A***, Heparinase II and III treatment of media Nluc-GPC4 reduced the binding of GPC4 to DEAE anion exchange column, by removing the negatively charged heparan sulfate side chains (one-sample *t* test *p* = 0.0365, Cohen’s *d* = 3.482). ***B***, The surface Nluc-GPC4 released by PI-PLC showed similar binding to DEAE as basal release GPC4, indicating that both lipase and protease release mechanisms contain the heparan sulfate modification and are capable of known synaptogenic signaling functions (*t* test *p* = 0.9467, Cohen’s *d* = 0.390); *p < 0.05, ns: not significant.

Heparan sulfate attachment to GPC4 plays a critical role in downstream signaling, including known synaptogenic functions through RPTPs and GPR158 (Condomitti et al., 2018; Farhy-Tselnicker et al., 2017). Unlike GPI-anchor cleavage, which releases full-length protein, proteolytic shedding could generate multiple extracellular fragments. Thus, it is critical to determine whether the released GPC4 contains heparan sulfate attachment sites after proteolytic shedding. To test heparan sulfate attachment to released GPC4, we performed a pulldown assay with an anion exchange column, which binds to the negative charge of heparan sulfate chain (Kojima et al., 1992). As a positive control, we tested whether the surface GPC4 contains heparan sulfate attachment. Treatment of basal condition medium with heparanases reduced the binding of nluc-GPC4 to the column (mean difference 95% CI = −1.091 to −0.3206, Cohen’s *d* = 4.153, one-sample *t* test *p* = 0.0365; Fig. 5*A*), indicating that DEAE binding of GPC4 requires heparan sulfate attachment. Interestingly, the released GPC4 in the basal condition shows a similar binding affinity as that acutely released from the cell surface by PI-PLC (mean difference 95% CI = −0.2504–0.2181, Cohen’s *d* = 0.3909, *p* = 0.9467; Fig. 5*B*). These results show that constitutively shed Nluc-GPC4 from astrocytes contains the heparan sulfate modification, indicating that the cleavage site of GPC4 is located downstream of heparan sulfate attachment sites. Overall, the released GPC4 from astrocytes by either mode contains the heparan sulfate posttranslational modification, which is required for its downstream signaling for synapse development.

## Discussion

Here, we show that GPC4 is expressed on the surface membrane via GPI-anchorage in astrocytes. Our results show that GPC4 is robustly released from the astrocyte surface predominantly by a proteolytic and partially by a lipase shedding mechanism. Soluble GPC4 released by either mode contains the heparan sulfate posttranslational modification, which is necessary for GPC4 synaptogenic function. Further, our data identify ADAM9 as a protease that releases GPC4 from astrocytes.

GPC4 was identified as an astrocyte-derived signaling factor. The released GPC4 was shown to induce synapse formation and maturation in cultured neurons (Allen et al., 2012). Further study of astrocyte secreted GPC4 function identified a heparan sulfate-dependent interaction between GPC4 and RPTPσ and RPTPδ to drive neuronal pentraxin 1 (NP1)-mediated recruitment of AMPA receptors (Farhy-Tselnicker et al., 2017). GPC4 is also expressed in neurons and presynaptic GPC4 has been found to play a role in the construction of mossy fiber synapses in the hippocampus via postsynaptic GPR158 (Condomitti et al., 2018). Given the observed roles of GPC4 in both a secreted form in astrocytes and in a cell membrane-tethered form in neurons, the release of GPC4 may play a distinct role in synaptogenesis depending on the cell type. For example, the release of GPC4 from astrocytes may facilitate the maturation of nearby synapses in a non-cell autonomous manner. Alternatively, release of GPC4 from the presynaptic membrane may inhibit the synaptic adhesion of a specific type of synapse, such as hippocampal mossy fiber synapses. Thus, elucidating the regulatory mechanism of surface GPC4 is critical to understand the physiological roles of GPC4 in synapse development.

To study the mechanism of GPC4 shedding, we found that it was necessary to develop a new tool to counter the poor antigenicity of GPC4. We developed a Nluc-based GPC4 release assay, which results in a quantitative, sensitive, and non-destructive assay capable of examining the temporal dynamics of GPC4 release. By using this method, we are able to distinguish the different modes of GPC4 release.

Biotinylation assays showed that GPC4 is released from the cell surface, not from the internal pool in the secretory pathway. It is notable that several GPI-anchored proteins are released with a lipid tail-intact form via lipid-associated particles such as on an extracellular vesicle or exosome (Muller, 2018). For example, exosomal GPC1 level is elevated in the serum of patients with pancreatic cancer and is associated with poor prognosis (Melo et al., 2015; Zhou et al., 2018). However, our results show that vesicular release is not a major route of GPC4 release from astrocytes and the majority of released GPC4 exists as a soluble form.

The soluble released form of GPC4 is generated largely by proteolytic shedding, which is partially blocked by GM6001, a pan-metalloprotease inhibitor. Our TIMP overexpression and *shRNA* screens indicated that ADAM9 in part mediates the shedding of GPC4 in astrocytes. However, the magnitude of reduction is limited to 20–30%, which suggests that there are other proteases working in parallel. Based on our GM6001 results, we expect these proteases to be unaffected by zinc-dependent proteolytic mechanisms, such as serine proteases like the (TMPRSS) family, which are not inhibited by GM6001 (Lam et al., 2015).

Our data show that ADAM9 overexpression is capable of shedding GPC4 from astrocytes. It is possible that ADAM9 induces GPC4 shedding through action on intermediate substrates or activation of an intermediate protease. For example, ADAM9 plays roles in APP shedding through regulation of surface expression and activity of ADAM10 by acting as an ADAM10 sheddase (Moss et al., 2011). We specifically examined the ability of ADAM10 to shed GPC4, and found that neither overexpression nor *shRNA* knock-down of *Adam10* is sufficient to impact GPC4 shedding in astrocytes. Based on these results, we favor the interpretation that GPC4 is a direct substrate of ADAM9 but do not rule out intermediate actors in this shedding mechanism.

AT pulldown assays showed that ∼30% of soluble GPC4 in the medium contains the core structure of the GPI-anchor, indicating a GPI lipase activity on GPC4 in astrocytes. There are several families of GPI-anchor lipases that releases GPI-anchored proteins. The glycerophosphodiester phosphodiesterase (GDPD) family and GPLD1 catalyze phospholipase-C (PLC) and PLD cleavage of the GPI-anchor, respectively (Hettmann et al., 2003; Park et al., 2013; van Veen et al., 2017). Overexpression of GDE2/GDPD5 in neuroblastoma and SH-SY5Y neuronal cells facilitates the release of GPC6 and decreases the surface level of GPC6 (Matas-Rico et al., 2016; Salgado-Polo et al., 2020). Knock-down or knock-out of *Gpc6* induces neuronal differentiation similarly to the overexpression of GDE2, suggesting that removal of surface GPC6 by GDE2 mediates the neuronal differentiation in a cell-autonomous manner. Interestingly, a recent study showed that the plasma level of GPLD1 increases after exercise (Horowitz et al., 2020). It is notable that overexpression of GPLD1 WT but not a catalytic-dead mutant ameliorates age-related cognitive impairment and enhances adult neurogenesis in aged animals. These results underscore the significant roles of the GPI-anchored proteins and their regulation in neuronal development and function. The GPI lipase that mediates GPC4 release in astrocytes remains to be determined.

The synaptogenic function of GPC4 requires the presence of heparan sulfate side chain. These heparan sulfate side chains are attached to residues proximal to the C terminus and are thought to mediate binding to GPC4 receptors (Allen et al., 2012; Farhy-Tselnicker et al., 2017; Condomitti et al., 2018). While a lipase shedding mechanism releases GPC4 intact, including the heparan sulfate modification, proteolytic shedding releases a prodomain fragment, and depending on the cleavage site, could shed GPC4 with or without heparan sulfate modification. Our results show that endogenously shed GPC4 is pulled down by a DEAE anion exchange column at similar ratios as PI-PLC lipase shed GPC4, suggesting that endogenously shed GPC4 is heparan sulfate modified and capable of known synaptogenic functions.

Given our results, we expect both lipase and protease shed GPC4 to be capable of functioning as a synaptogenic factor. However, this raises questions as to why there are multiple shedding mechanisms for GPC4 and whether the GPI anchor posttranslational modification plays a role in, or modifies GPC4 function. There could be differences in stability between lipase and protease shed GPC4 in the extracellular space. For example, unlike an ectodomain fragment shed by a protease, the core GPI structure attached to the C terminus of the protein released by a GPI lipase may play a protective role against extracellular proteases, such as carboxypeptidase E, which is secreted by astrocytes (Ji et al., 2017; Klein et al., 1992). It is also possible that the location and the upstream regulatory signals may differ between proteolytic and GPI lipase enzymes, allowing for differential control of shedding. In addition, given the heterogeneity of astrocyte subpopulations, these activities may be achieved by distinct astrocyte subtypes (John Lin et al., 2017). Future investigations will be required to determine the localization, regulation, and subtype-specific expression of the releasing enzymes.

Overall, our study demonstrates that soluble forms of GPC4 are released from the astrocyte surface and contain the heparan sulfate side chain. ADAM9 is a key enzyme that mediates the release of GPC4 from astrocytes. Since, unlike other secreted factors, GPC4 is released from the cell surface by releasing enzymes, our study will provide an opportunity in understanding the regulatory mechanism by which astrocytes promotes synapse maturation and function.

## Synthesis

Reviewing Editor: Nathalie Rouach, College of France Decisions are customarily a result of the Reviewing Editor and the peer reviewers coming together and discussing their recommendations until a consensus is reached. When revisions are invited, a fact-based synthesis statement explaining their decision and outlining what is needed to prepare a revision will be listed below. The following reviewer(s) agreed to reveal their identity: Alyson Fournier.

The current consensus is that the manuscript provides interesting novel data regarding the mechanisms of astrocyte secretion of glypican 4. The main criticism is that the shRNA experiments are not well controlled and this should be addressed. In addition, since several mechanisms are here identified, the authors should edit the text to put less emphasis on Adam9 as the major enzyme.

Reviewer 1

The authors address a novel mechanism of glypican-4 processing.

Astrocytes are known to produce factors including glypican-4 (GPC4) that regulate synaptogenesis and synapse maturation. Post translational processing of proteins can play an important role in regulating the activity of proteins and here the authors explore the role of cell surface cleavage of the GPI-anchored GPC4. In this manuscript the authors demonstrate that GPC4 is cleaved from the surface of astrocytes through an ADAM9-dependent mechanism.

The data is of high quality but is limited to the biochemical characterization of the cleavage of GPC4 without any follow-up to describe the functional consequences of this cleavage and in this sense the manuscript is rather limited in scope. The major weakness of the manuscript is that the shRNA experiments are not sufficiently controlled.

1. The Adam9shRNA experiment is lacking evidence to demonstrate that Adam9 is expressed in astrocytes and that its expression is suppressed by the shRNA. Further, multiple shRNA constructs should be shown to have the same effect or rescue experiments with an shRNA-resistant Adam9 construct should be performed.

2. The authors provide evidence that GPC4 is anchored to the cell membrane of 293T cells and astrocytes by exhibiting its sensitivity to PI-PLC-dependent release into the conditioned media. The cleavage from astrocytes would be more convincing if a non-GPI anchored protein was probed to demonstrate insensitivity to the PI-PLC treatment.

Minor Comments

3. The experiments do not demonstrate that GPC4 is directly targeted by ADAM9 and the authors should be careful in their interpretation. This issue is clearly addressed in the discussion but is vague in the results section.

4. Line 228 - “Interestingly, PI-PLC treatment also slightly increased the level of TSP1 in the culture medium, indicating that a portion of secreted TSP1 is associated with GPI-anchored proteins on the astrocyte surface.” TSP-1 is not introduced in the manuscript and the significance of this observation is unclear.

Reviewer 2

Regulation of synapse formation by astrocyte secreted factors is crucial for normal brain function. This manuscript studies the mechanisms of astrocyte secretion of one such factor, glypican 4 (Gpc4), a member of GPI-anchored proteoglycans family. Authors show that Gpc4 is cleaved from the astrocytes surface by GPI anchor release, and by proteolytic shedding, and identify the enzyme partially responsible for the shedding mechanism. This work elucidates the unknown mechanisms of Gpc4 secretion and provides important insight for future in vitro and in vivo studies that focus on Gpc4-mediated synapse regulation.

This is a very nice paper showing how the astrocyte-expressed synapse regulating protein glypican 4 (Gpc4) is secreted from the astrocytes. The authors use in vitro cell cultures and biochemical methods, including a luciferase reporter construct, to study the mechanisms / enzymes that mediate Gpc4 release. Authors find that Gpc4 is cleaved from the cell surface by different mechanisms, including GPI anchor cleavage and proteolytic shedding. Authors further identify Adam9 as one of the enzymes responsible for Gpc4 shedding. Finally, authors show that soluble Gpc4 released by either mechanism, contains the HS chains, which are necessary for its effect on synapse formation. These data show the existing diversity of mechanisms that regulate Gpc4 release, and that the secreted Gpc4 is a mixture of cleaved products, a very interesting finding. These data further provide important mechanistic insight for future studies that focus on Gpc4-mediated synapse formation in different cell types and/ or brain regions. Overall, the manuscript is well written and data presented clearly. I have several comments as outlined below:

1. The effect of Adam9 knock down on Gpc4 shedding is mild. Could that be due to insufficient shRNA knock down? It would be helpful to note this possibility in the text or add data showing Adam9 shRNA knock down efficiency.

2. The title suggests proteolytic shedding to be the predominant way Gpc4 is released, however, the data in the manuscript show that at least ∼40% of Gpc4 is GPI-cleaved (Fig3C). That is quite a large portion of the total released protein. It would be more reflective of the findings to modify the title to say that both mechanisms exist for Gpc4 release, with shedding being a bit more dominant.

3. It would be helpful to add the p values to the plots in each figure.

4. Fig1, Fig2D-E - numbers of biological replicates are missing.

## References

[B1] Allen NJ, Bennett ML, Foo LC, Wang GX, Chakraborty C, Smith SJ, Barres BA (2012) Astrocyte glypicans 4 and 6 promote formation of excitatory synapses via GluA1 AMPA receptors. Nature 486:410–414. 10.1038/nature11059 22722203 PMC3383085

[B2] Bajor M, Kaczmarek L (2013) Proteolytic remodeling of the synaptic cell adhesion molecules (CAMs) by metzincins in synaptic plasticity. Neurochem Res 38:1113–1121. 10.1007/s11064-012-0919-6 23124395 PMC3653053

[B3] Brew K, Nagase H (2010) The tissue inhibitors of metalloproteinases (TIMPs): an ancient family with structural and functional diversity. Biochim Biophys Acta 1803:55–71. 10.1016/j.bbamcr.2010.01.003 20080133 PMC2853873

[B4] Cahoy JD, Emery B, Kaushal A, Foo LC, Zamanian JL, Christopherson KS, Xing Y, Lubischer JL, Krieg PA, Krupenko SA, Thompson WJ, Barres BA (2008) A transcriptome database for astrocytes, neurons, and oligodendrocytes: a new resource for understanding brain development and function. J Neurosci 28:264–278. 10.1523/JNEUROSCI.4178-07.2008 18171944 PMC6671143

[B5] Chen R, Walter EI, Parker G, Lapurga JP, Millan JL, Ikehara Y, Udenfriend S, Medof ME (1998) Mammalian glycophosphatidylinositol anchor transfer to proteins and posttransfer deacylation. Proc Natl Acad Sci USA 95:9512–9517. 10.1073/pnas.95.16.9512 9689111 PMC21369

[B6] Choi BR, Dobrowolski M, Sockanathan S (2021) GDE2 expression in oligodendroglia regulates the pace of oligodendrocyte maturation. Dev Dyn 250:513–526. 10.1002/dvdy.265 33095500 PMC8083128

[B7] Christopherson KS, Ullian EM, Stokes CC, Mullowney CE, Hell JW, Agah A, Lawler J, Mosher DF, Bornstein P, Barres BA (2005) Thrombospondins are astrocyte-secreted proteins that promote CNS synaptogenesis. Cell 120:421–433. 10.1016/j.cell.2004.12.020 15707899

[B8] Condomitti G, Wierda KD, Schroeder A, Rubio SE, Vennekens KM, Orlandi C, Martemyanov KA, Gounko NV, Savas JN, de Wit J (2018) An input-specific orphan receptor GPR158-HSPG interaction organizes hippocampal mossy fiber-CA3 synapses. Neuron 100:201–215.e9. 10.1016/j.neuron.2018.08.038 30290982 PMC6351853

[B9] Davis S, Aldrich TH, Ip NY, Stahl N, Scherer S, Farruggella T, DiStefano PS, Curtis R, Panayotatos N, Gascan H (1993) Released form of CNTF receptor alpha component as a soluble mediator of CNTF responses. Science 259:1736–1739. 10.1126/science.7681218 7681218

[B10] Dobrowolski M, Cave C, Levy-Myers R, Lee C, Park S, Choi BR, Xiao B, Yang W, Sockanathan S (2020) GDE3 regulates oligodendrocyte precursor proliferation via release of soluble CNTFRalpha. Development 147:dev180695. 10.1242/dev.180695 31932351 PMC6983723

[B11] Dowling C, Allen NJ (2018) Mice lacking glypican 4 display juvenile hyperactivity and adult social interaction deficits. Brain Plast 4:197–209. 10.3233/BPL-180079 30598870 PMC6311356

[B12] Farhy-Tselnicker I, van Casteren ACM, Lee A, Chang VT, Aricescu AR, Allen NJ (2017) Astrocyte-secreted glypican 4 regulates release of neuronal pentraxin 1 from axons to induce functional synapse formation. Neuron 96:428–445.e13. 10.1016/j.neuron.2017.09.053 29024665 PMC5663462

[B13] Feitsma K, Hausser H, Robenek H, Kresse H, Vischer P (2000) Interaction of thrombospondin-1 and heparan sulfate from endothelial cells. Structural requirements of heparan sulfate. J Biol Chem 275:9396–9402. 10.1074/jbc.275.13.9396 10734084

[B14] Fernández-Messina L, Ashiru O, Boutet P, Agüera-González S, Skepper JN, Reyburn HT, Valés-Gómez M (2010) Differential mechanisms of shedding of the glycosylphosphatidylinositol (GPI)-anchored NKG2D ligands. J Biol Chem 285:8543–8551. 10.1074/jbc.M109.045906 20080967 PMC2838276

[B15] Filmus J, Capurro M, Rast J (2008) Glypicans. Genome Biol 9:224. 10.1186/gb-2008-9-5-224 18505598 PMC2441458

[B16] Greco TM, Seeholzer SH, Mak A, Spruce L, Ischiropoulos H (2010) Quantitative mass spectrometry-based proteomics reveals the dynamic range of primary mouse astrocyte protein secretion. J Proteome Res 9:2764–2774. 10.1021/pr100134n 20329800 PMC2866110

[B17] Grell M, Douni E, Wajant H, Löhden M, Clauss M, Maxeiner B, Georgopoulos S, Lesslauer W, Kollias G, Pfizenmaier K, Scheurich P (1995) The transmembrane form of tumor necrosis factor is the prime activating ligand of the 80 kDa tumor necrosis factor receptor. Cell 83:793–802. 10.1016/0092-8674(95)90192-2 8521496

[B18] Heise K, Oppermann H, Meixensberger J, Gebhardt R, Gaunitz F (2013) Dual luciferase assay for secreted luciferases based on Gaussia and NanoLuc. Assay Drug Dev Technol 11:244–252. 10.1089/adt.2013.509 23679848

[B19] Hettmann T, Siddiqui RA, von Langen J, Frey C, Romão MJ, Diekmann S (2003) Mutagenesis study on the role of a lysine residue highly conserved in formate dehydrogenases and periplasmic nitrate reductases. Biochem Biophys Res Commun 310:40–47. 10.1016/j.bbrc.2003.08.114 14511645

[B20] Hong Y, Ohishi K, Inoue N, Kang JY, Shime H, Horiguchi Y, van der Goot FG, Sugimoto N, Kinoshita T (2002) Requirement of N-glycan on GPI-anchored proteins for efficient binding of aerolysin but not Clostridium septicum alpha-toxin. EMBO J 21:5047–5056. 10.1093/emboj/cdf508 12356721 PMC129030

[B21] Horowitz AM, Fan X, Bieri G, Smith LK, Sanchez-Diaz CI, Schroer AB, Gontier G, Casaletto KB, Kramer JH, Williams KE, Villeda SA (2020) Blood factors transfer beneficial effects of exercise on neurogenesis and cognition to the aged brain. Science 369:167–173. 10.1126/science.aaw2622 32646997 PMC7879650

[B22] Ji L, Wu HT, Qin XY, Lan R (2017) Dissecting carboxypeptidase E: properties, functions and pathophysiological roles in disease. Endocr Connect 6:R18–R38. 10.1530/EC-17-0020 28348001 PMC5434747

[B23] John Lin CC, Yu K, Hatcher A, Huang TW, Lee HK, Carlson J, Weston MC, Chen F, Zhang Y, Zhu W, Mohila CA, Ahmed N, Patel AJ, Arenkiel BR, Noebels JL, Creighton CJ, Deneen B (2017) Identification of diverse astrocyte populations and their malignant analogs. Nat Neurosci 20:396–405. 10.1038/nn.4493 28166219 PMC5824716

[B24] Kato M, Wang H, Kainulainen V, Fitzgerald ML, Ledbetter S, Ornitz DM, Bernfield M (1998) Physiological degradation converts the soluble syndecan-1 ectodomain from an inhibitor to a potent activator of FGF-2. Nat Med 4:691–697. 10.1038/nm0698-691 9623978

[B25] Klein RS, Das B, Fricker LD (1992) Secretion of carboxypeptidase E from cultured astrocytes and from AtT-20 cells, a neuroendocrine cell line: implications for neuropeptide biosynthesis. J Neurochem 58:2011–2018. 10.1111/j.1471-4159.1992.tb10941.x 1573389

[B26] Kojima T, Leone CW, Marchildon GA, Marcum JA, Rosenberg RD (1992) Isolation and characterization of heparan sulfate proteoglycans produced by cloned rat microvascular endothelial cells. J Biol Chem 267:4859–4869. 10.1016/S0021-9258(18)42910-9 1537864

[B27] Konoshenko MY, Lekchnov EA, Vlassov AV, Laktionov PP (2018) Isolation of extracellular vesicles: general methodologies and latest trends. Biomed Res Int 2018:8545347. 10.1155/2018/8545347 29662902 PMC5831698

[B28] Lam DK, Dang D, Flynn AN, Hardt M, Schmidt BL (2015) TMPRSS2, a novel membrane-anchored mediator in cancer pain. Pain 156:923–930. 10.1097/j.pain.0000000000000130 25734995 PMC5215063

[B29] Li Q, Cheng Z, Zhou L, Darmanis S, Neff NF, Okamoto J, Gulati G, Bennett ML, Sun LO, Clarke LE, Marschallinger J, Yu G, Quake SR, Wyss-Coray T, Barres BA (2019) Developmental heterogeneity of microglia and brain myeloid cells revealed by deep single-cell RNA sequencing. Neuron 101:207–223.e10. 10.1016/j.neuron.2018.12.006 30606613 PMC6336504

[B101] Lichtenthaler SF, Lemberg MK, Fluhrer R (2018) Proteolytic ectodomain shedding of membrane proteins in mammals-hardware, concepts, and recent developments. EMBO J 37:e99456. 29976761 10.15252/embj.201899456 PMC6068445

[B30] Linsenmeier L, Mohammadi B, Wetzel S, Puig B, Jackson WS, Hartmann A, Uchiyama K, Sakaguchi S, Endres K, Tatzelt J, Saftig P, Glatzel M, Altmeppen HC (2018) Structural and mechanistic aspects influencing the ADAM10-mediated shedding of the prion protein. Mol Neurodegener 13:18. 10.1186/s13024-018-0248-6 29625583 PMC5889536

[B31] Matas-Rico E, van Veen M, Leyton-Puig D, van den Berg J, Koster J, Kedziora KM, Molenaar B, Weerts MJA, de Rink I, Medema RH, Giepmans BNG, Perrakis A, Jalink K, Versteeg R, Moolenaar WH (2016) Glycerophosphodiesterase GDE2 promotes neuroblastoma differentiation through glypican release and is a marker of clinical outcome. Cancer Cell 30:548–562. 10.1016/j.ccell.2016.08.016 27693046

[B32] Melo SA, Luecke LB, Kahlert C, Fernandez AF, Gammon ST, Kaye J, LeBleu VS, Mittendorf EA, Weitz J, Rahbari N, Reissfelder C, Pilarsky C, Fraga MF, Piwnica-Worms D, Kalluri R (2015) Glypican-1 identifies cancer exosomes and detects early pancreatic cancer. Nature 523:177–182. 10.1038/nature14581 26106858 PMC4825698

[B33] Mollinedo F, Gajate C, Martín-Santamaría S, Gago F (2004) ET-18-OCH3 (edelfosine): a selective antitumour lipid targeting apoptosis through intracellular activation of Fas/CD95 death receptor. Curr Med Chem 11:3163–3184. 10.2174/0929867043363703 15579006

[B34] Moss ML, Powell G, Miller MA, Edwards L, Qi B, Sang QXA, De Strooper B, Tesseur I, Lichtenthaler SF, Taverna M, Zhong JL, Dingwall C, Ferdous T, Schlomann U, Zhou P, Griffith LG, Lauffenburger DA, Petrovich R, Bartsch JW (2011) ADAM9 inhibition increases membrane activity of ADAM10 and controls α-secretase processing of amyloid precursor protein. J Biol Chem 286:40443–40451. 10.1074/jbc.M111.280495 21956108 PMC3220463

[B35] Muller GA (2018) The release of glycosylphosphatidylinositol-anchored proteins from the cell surface. Arch Biochem Biophys 656:1–18. 10.1016/j.abb.2018.08.009 30120921

[B36] Nagappan-Chettiar S, Johnson-Venkatesh EM, Umemori H (2017) Activity-dependent proteolytic cleavage of cell adhesion molecules regulates excitatory synaptic development and function. Neurosci Res 116:60–69. 10.1016/j.neures.2016.12.003 27965136 PMC5376514

[B37] Park S, Lee C, Sabharwal P, Zhang M, Meyers CL, Sockanathan S (2013) GDE2 promotes neurogenesis by glycosylphosphatidylinositol-anchor cleavage of RECK. Science 339:324–328. 10.1126/science.1231921 23329048 PMC3644959

[B38] Reiss K, Maretzky T, Ludwig A, Tousseyn T, de Strooper B, Hartmann D, Saftig P (2005) ADAM10 cleavage of N-cadherin and regulation of cell-cell adhesion and beta-catenin nuclear signalling. EMBO J 24:742–752. 10.1038/sj.emboj.7600548 15692570 PMC549617

[B39] Salgado-Polo F, van Veen M, van den Broek B, Jalink K, Leyton-Puig D, Perrakis A, Moolenaar WH, Matas-Rico E (2020) Sequence-dependent trafficking and activity of GDE2, a GPI-specific phospholipase promoting neuronal differentiation. J Cell Sci 133:jcs235044. 10.1242/jcs.235044 31932507 PMC7033719

[B40] Sanz RL, Ferraro GB, Kacervosky J, Salesse C, Gowing E, Hua L, Rambaldi I, Beaubien F, Holmbeck K, Cloutier JF, Lévesque M, Murai K, Fournier AE (2018) MT3-MMP promotes excitatory synapse formation by promoting Nogo-66 receptor ectodomain shedding. J Neurosci 38:518–529. 10.1523/JNEUROSCI.0962-17.2017 29196321 PMC6596192

[B42] Shin DJ, Cho D, Kim YR, Rhee JH, Choy HE, Lee JJ, Hong Y (2006) Diagnosis of paroxysmal nocturnal hemoglobinuria by fluorescent clostridium septicum alpha toxin. J Mol Microbiol Biotechnol 11:20–27. 10.1159/000092816 16825787

[B43] van Veen M, Matas-Rico E, van de Wetering K, Leyton-Puig D, Kedziora KM, De Lorenzi V, Stijf-Bultsma Y, van den Broek B, Jalink K, Sidenius N, Perrakis A, Moolenaar WH (2017) Negative regulation of urokinase receptor activity by a GPI-specific phospholipase C in breast cancer cells. Elife 6:e23649. 10.7554/eLife.23649 28849762 PMC5576486

[B44] Vidal M (2020) Exosomes and GPI-anchored proteins: judicious pairs for investigating biomarkers from body fluids. Adv Drug Deliv Rev 161–162:110–123. 10.1016/j.addr.2020.08.006 32828789

[B102] Webber CA, Hocking JC, Yong VW, Stange CL, McFarlane S (2002) Metalloproteases and guidance of retinal axons in the developing visual system. J Neurosci 22:8091–8100. 12223563 10.1523/JNEUROSCI.22-18-08091.2002 PMC6758082

[B45] Wilson ES, Newell-Litwa K (2018) Stem cell models of human synapse development and degeneration. Mol Biol Cell 29:2913–2921. 10.1091/mbc.E18-04-0222 30475098 PMC6329912

[B46] Zhang Y, Chen K, Sloan SA, Bennett ML, Scholze AR, O’Keeffe S, Phatnani HP, Guarnieri P, Caneda C, Ruderisch N, Deng S, Liddelow SA, Zhang C, Daneman R, Maniatis T, Barres BA, Wu JQ (2014) An RNA-sequencing transcriptome and splicing database of glia, neurons, and vascular cells of the cerebral cortex. J Neurosci 34:11929–11947. 10.1523/JNEUROSCI.1860-14.2014 25186741 PMC4152602

[B47] Zhou CY, Dong YP, Sun X, Sui X, Zhu H, Zhao YQ, Zhang YY, Mason C, Zhu Q, Han SX (2018) High levels of serum glypican-1 indicate poor prognosis in pancreatic ductal adenocarcinoma. Cancer Med 7:5525–5533. 10.1002/cam4.1833 30358133 PMC6246926

