# Supplementary figures and images for: Heparan Sulfated Glypican-4 Is Released from Astrocytes by Proteolytic Shedding and GPI-Anchor Cleavage Mechanisms
Source: eNeuro. 2021 Aug 6;8(4):ENEURO.0069-21.2021. doi: 10.1523/ENEURO.0069-21.2021 (PMC8387153; doi:10.1523/ENEURO.0069-21.2021)

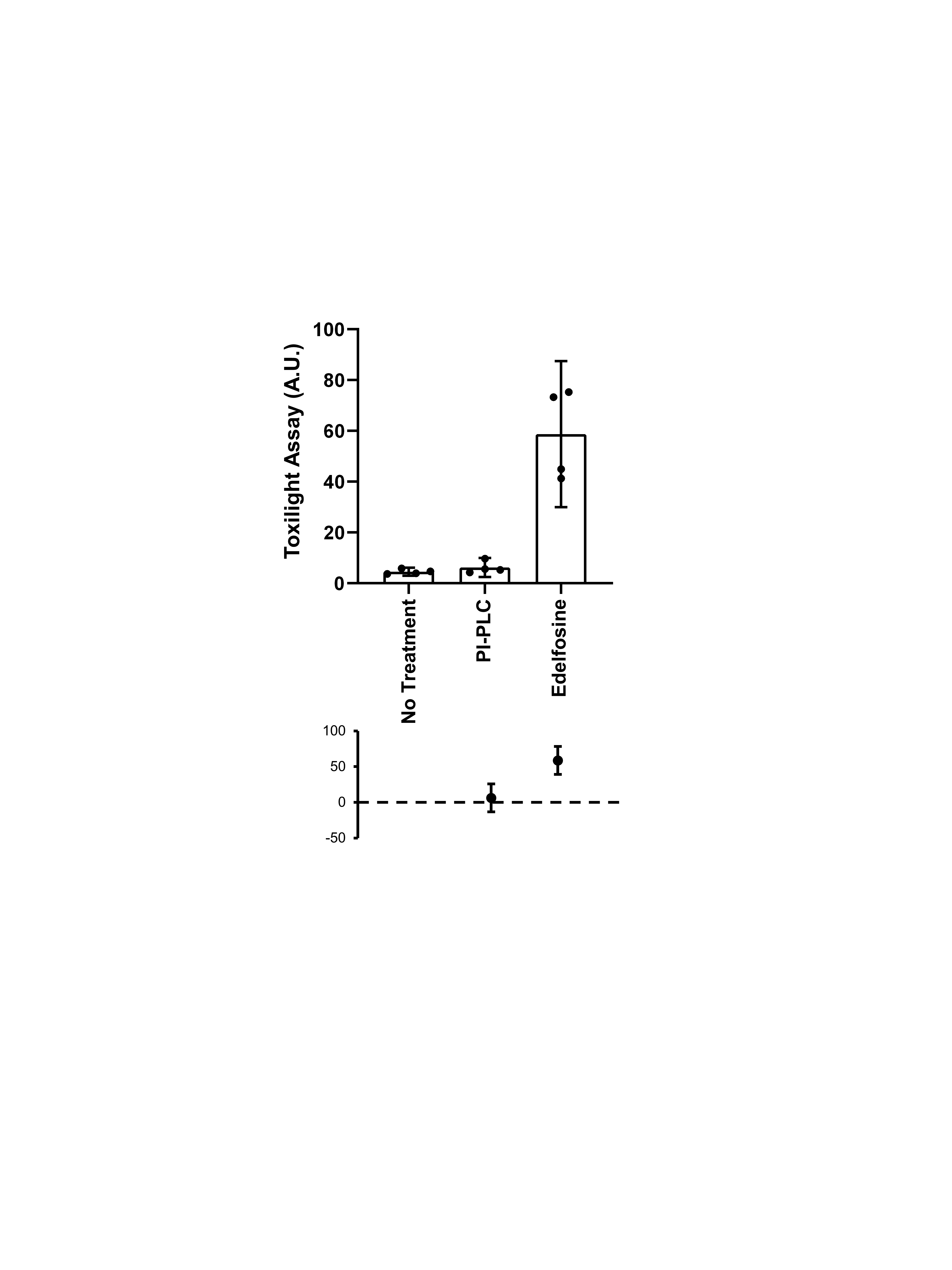

Supplement: Extended Data Figure 1-1 — Extended data supporting Figure 1C. Cell toxicity assay of WT astrocytes with and without overnight PI-PLC treatment. Higher levels of bioluminescence indicate a loss of cell integrity and leakage of intracellular adenylate kinase into the media. Edelfosine treatment (50 μm, 1 h) is included as a positive control for cell death. Tukey’s multiple comparison Ctrl versus PI-PLC p = 0.964, Cohen’s d = 0.907. Tukey’s multiple comparison Ctrl versus Edelfosine p < 0.0001, Cohen’s d = 2.870. Download Figure 1-1, TIF file. [file enu-eN-NWR-0069-21-s03.tif]

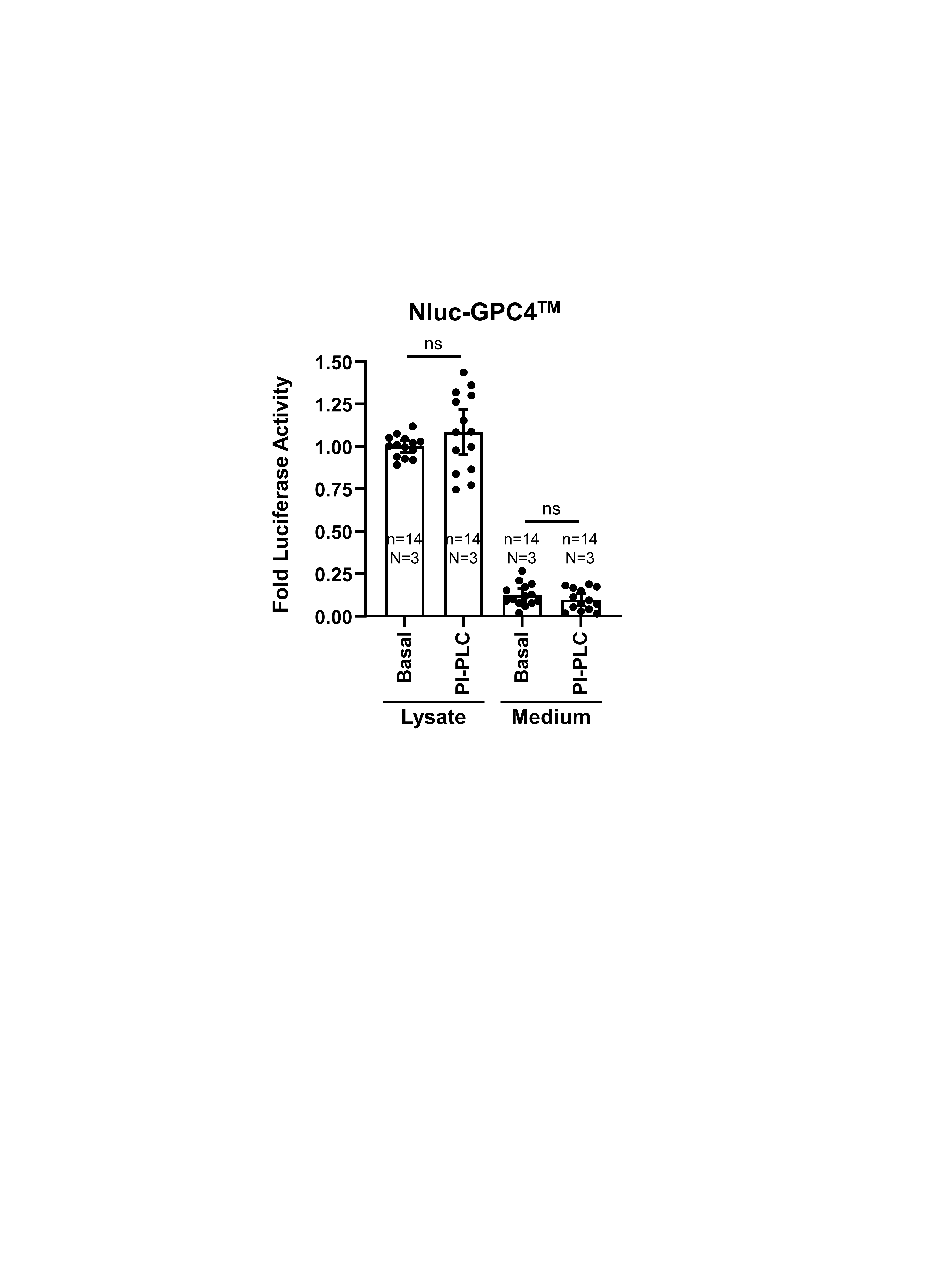

Supplement: Extended Data Figure 2-1 — Extended data supporting Figure 2D. Astrocytes expressing Nluc-GPC4-TM was incubated in fresh media with and without PI-PLC for 3 h, and Nluc signal was measured in the cell lysate and media. Nluc signal was normalized to untreated lysate conditions for each biological replicate. PI-PLC treatment did not result in the release of GPC4-TM from astrocytes. Lysate +/– PI-PLC unpaired t test p = 0.19, Cohen’s d = 0.49. Media +/– PI-PLC unpaired t test p = 0.27, Cohen’s d = 0.50. Download Figure 2-1, TIF file. [file enu-eN-NWR-0069-21-s04.tif]
